# Altered motor coordination, vocal communication, and cerebellar circuit connectivity in mice carrying a near-complete human chromosome 21

**DOI:** 10.1038/s41398-025-03744-2

**Published:** 2025-11-22

**Authors:** Rachel Stander, Nithilah Ayyappan, Darek Sikorski, Meike E. van der Heijden, Kuangfu Hsiao

**Affiliations:** 1 Children’s National Hospital, Washington, DC 20010 USA; 2 Department of Neuroscience, University of Colorado Boulder, Boulder, CO 80309 USA; 3 University of North Carolina, Chapel Hill, NC USA; 4 Fralin Biomedical Research Institute, Virginia Tech Carilion, Roanoke, VA 24016 USA; 5 School of Neuroscience, Virginia Tech, Blacksburg, VA 24061 USA; 6 Department of Pediatrics, Virginia Tech Carilion, Roanoke, VA 24016 USA; 7 George Washington University School of Medicine and Health Sciences, Washington, DC 20052 USA

**Keywords:** Learning and memory, Autism spectrum disorders

## Abstract

Individuals with Down syndrome (DS) frequently face challenges with motor control and coordination, affecting their daily physical movements. The neural mechanism underlying motor coordination deficits in DS remains poorly understood. Using the TcMAC21 mice, which carry an extra nearly complete human chromosome 21 in addition to two copies of mouse orthologs, we characterized altered motor function and identified cerebellar circuit dysfunction underlying motor adaptation deficits. We also revealed disrupted Purkinje neuron organization and hypertrophied synapses from climbing fiber afferents, accompanied by specific deficits in cerebellar-dependent behaviors, including motor learning, vocalizations, and maternal care. In vivo calcium recordings showed stochastic decoupling of cerebellar nuclear activity from locomotion states, while cerebello-thalamic synchrony was reduced. Selective elevation of intracellular calcium in developing Purkinje neurons recapitulates motor adaptation deficits and climbing fiber phenotype observed in the TcMAC21 model, supporting the conclusion that cell-autonomous calcium signaling is a functionally relevant feature. This study provides a framework for understanding both motor and cerebellar deficits in DS, extending beyond cortico-centric models.

## Introduction

Down syndrome (DS) results from an extra copy of human chromosome 21 (HSA21) and, in addition to causing intellectual disability, significantly impacts motor skills and speech, thereby affecting independent living and communication [1, 2]. Hypoplasia of the cerebellum is consistently observed in both individuals with DS and mouse models [3, 4]. The convergence of these prominent symptoms in DS, along with growing evidence implicating dysfunction in this brain region in motor, speech, and social impairments [5–7], suggests that impaired function of this structure may underlie these deficits.

The olivocerebellar circuit, particularly climbing fiber inputs to Purkinje neurons, plays a critical role in motor learning and coordination. These afferents, originating in the inferior olive, are topographically organized into parasagittal zones [8, 9] and drive synaptic plasticity in Purkinje neurons [10, 11]. The climbing fiber signals can shape cerebellar nuclear output, forming a precisely timed circuit essential for motor learning and coordination, by mediating complex spikes in Purkinje neurons through strong glutamatergic inputs from climbing fiber synapses [12, 13]. Disruption of olivocerebellar signaling impairs the ability of the cerebellum to adapt motor output in response to sensory prediction errors, a core mechanism for fine-tuning movement. Consequently, olivocerebellar dysfunction is predicted to manifest as deficits in cerebellar-dependent locomotor adaptation tasks, significantly impacting motor learning processes.

While generalized cerebellar hypoplasia and granule cell loss have long been recognized as hallmark features in DS [3,4,14], interventions aimed at correcting cerebellar volume deficits, such as sonic hedgehog (Shh) agonist treatments injected at the perinatal stage [15–17] or through granule cell-targeted viral expression [18], have yielded only partial functional recovery. The limited efficacy of interventions aimed solely at restoring cerebellar volume highlights that DS pathology extends beyond structural anomalies.

Beyond a gross reduction in cerebellar size, how HSA21 gene triplication disrupts cerebellar physiology remains undefined. Genes such as *PCP4*, *RCAN1*, and *DYRK1A* suppress the calcineurin pathway, compromising calcium buffering and synaptic function in Purkinje neurons [19–21]. This dysregulation can affect both parallel fiber and climbing fiber inputs, contributing to cerebellar learning deficits [22, 23].

Despite increasing knowledge of gene-level disruptions, a systems-level understanding of how calcium dysregulation impacts circuit function and behavior remains unknown. The TcMAC21 model provides an exceptional platform to bridge this gap through its comprehensive genetic representation of HSA21 [24–26], offering a unique opportunity to dissect how developmental molecular disruptions propagate through cerebellar circuits. We employed a multi-scale systems approach, integrating behavioral analysis with cellular morphology and network physiology, to trace how early molecular disruptions reshape circuit dynamics and ultimately impair behavioral output.

Our findings reveal that developmental calcium signaling alterations fundamentally reorganize cerebellar microcircuit architecture, disrupting the temporal precision of information flow from sensory input to motor output. These findings provide a mechanistic framework linking gene dosage to network-level dysfunction, and identify specific points of vulnerability that may serve as therapeutic entry points for restoring cerebellar function.

## Materials and methods

All experimental protocols were approved by the Institutional Animal Care and Use Committee (IACUC) at the Children’s National Medical Center.

### Mice

TcMAC21 mice (JAX #035561) and euploid littermate controls were maintained by breeding female TcMAC21 to male B6D2F1/J (JAX #100006) mice. The TcMAC21 strain, distributed by The Jackson Laboratory, was derived from RIKEN ID RBRC05796 STOCK Tc(HSA21q-MAC1) provided by the RIKEN BRC through the National BioResource Project of the MEXT, Japan [25]. *Pcp2*-Cre mice (JAX #010536) [27] were maintained on C57BL/6 J background. Breeder mice (6-8 weeks) were obtained from Jackson Laboratory. Experiments used animals from at least two litters per group. Mice were housed under a 12:12 light/dark cycle at 22 ± 2 °C with *ad libitum* food and water in standard polycarbonate cages with corn cob bedding and environmental enrichment. Both male and female mice were used unless otherwise specified. Ages and sample sizes for each experiment are detailed in individual behavioral sections.

### Animal behaviors

Animals were randomly assigned to experimental groups using a random number generator. Litter effects were minimized by including animals from at least two litters per experimental group. Sample sizes were determined based on effect sizes from prior literature and pilot experiments, with a minimum of n = 8-10 per group to detect medium effect sizes (Cohen’s d = 0.8) with 80% power at α = 0.05. Investigators were blinded to genotype during behavioral testing and data analysis where feasible. For experiments involving coat color differences (euploid vs. TcMAC21), complete blinding was not possible during testing but was maintained during data analysis.

#### ErasmusLadder analysis

Motor control performance to measure cerebellar function was assessed through daily testing of the ErasmusLadder task according to the procedure of Vinueza Veloz et al. [28], and was modified with test parameters as previously described [29]. Open field testing was performed as previously described [30].

#### Pup separation vocalization

Pup separation vocalizations were recorded by isolating pups (P8) individually in a soundproof chamber for 5 min as previously described [31]. Vocalizations were categorized by USVs (25-130 kHz). We quantified vocalization characteristics (mean frequency, range, duration, and tonality) using DeepSqueak’s automated calculations based on user-identified calls [32].

#### Pup retrieval test

Postpartum dams were given 24 h for nest building. On P3 and P5, three pups were placed in different corners of the home cage (30x45x15 cm) with the dam in the fourth corner. Tests occurred in the dark phase under red light as previously described [31]. Retrieval latency was recorded over 10 min, with a 600 s maximum score if unsuccessful.

#### Open field test

Exploratory activity and anxiety-like behavior of mice were examined in both male and female mice by the open field test as previously described [30]. Briefly, mice were transported to the testing room and allowed to acclimate for 15 min before each test. The testing room was illuminated with overhead lighting at ~450 lux. For the testing session, each mouse was gently placed in a corner of an open field Plexiglas clear chamber (21 cm × 21 cm × 30 cm) and allowed to move freely for 40 min. The data were collected using the open field activity monitoring system (AccuScan Instruments, Inc. Columbus, OH), which uses photocell emitters and receptors forming an x-y grid of invisible infrared beams.

#### Accelerated rotarod test

Each mouse was placed on a rotating bar, and the time the mouse could maintain balance while the rotation was accelerated from 5 rpm to the maximum speed (40 rpm) in 5 min was measured. The testing phase consisted of 6 consecutive days of one trial per day. Each trial was terminated when the mouse fell off, made one complete rotation without walking on the rotating rod, or reached 6 min (1 min passed the maximum speed). The latency to fall from the rotating rod was scored by automatic timers and falling sensors on the rotarod.

#### Active avoidance test with auditory cue

A two-way shuttle box (MED-APA-B1M; Med Associates, VT) was used to evaluate conditional learning of an aversive stimulus, and indirectly test hearing in female TcMAC21 mice. During each session, mice were initially placed in the dark box with the guillotine door open and allowed 5 min of free exploration. After which a pure tone conditioned stimulus (CS: 15 kHz, 71 dB SPL) was presented for 10 s, requiring the mouse to shuttle to the opposite chamber to avoid receiving an unconditioned stimulus (US: 0.36 mA, 2-second foot shock). Performance was measured across 50 trials per day (with inter-trial intervals of 30 ± 5 s) for six consecutive days, with learning quantified by the progressive increase in successful avoidance responses when presented with the auditory cue.

### Viral constructs

The Cre-inducible adeno-associated virus (AAV) vectors expressing hM3D DREADD with cilia-targeting-sequence (CTS), hM3D-CTS, were generated by subcloning into AAV.CAG-FLEX vector under the control of loxP sites. The cilia targeted hM3D-CTS DREADD and AAV.CAGFLEX were gifts from Drs. Gregory Pazour (University of Massachusetts Medical School) [33], Bryan Roth (University of North Carolina) [34], Chun-Li Zhang (RRID:Addgene_45560; RRID:Addgene_44361 ; RRID:Addgene_178583).

### Animal surgical procedures

Surgical procedures and viral injections were carried out under protocols approved by the Institutional Animal Care and Use Committee (IACUC) at Children’s National Medical Center.

#### Systematic intra-cisterna magna viral delivery and chemogenetic manipulation

Single Intra-Cisterna Magna (ICM) injections were performed as previously described in *Pcp2Cre* mouse pups. On postnatal day 2 (P2), we systematically delivered conditional viral constructs (AAV.CAG-FLEX-hM3D-CTS) to neonates via intra-cisterna magna (ICM) injection, which enhances cerebellar targeting while minimizing frontal cortex spread [35, 36]. Briefly, *Pcp2-Cre* neonates were cryoanesthetized and subsequently placed on a cold metal plate. A 30-gauge needle was used to pierce the skull 2 mm posterior to lambda at the midline, and 4 μl of AAV (AAV.CAG-FLEX-hM3D-CTS or AAV.CAG-FLEX-TdTomato) was injected into each cisterna magna (1.0E10 GC). AAV.CAG-FLEX-TdTomato and Custom AAV.CAG-FLEX-hM3D-CTS AAV production was carried out by Addgene and by Vigene Biosciences, respectively. Neonatal mice were kept with the parents until weaned. Mice were sacrificed at set time points as follows: 7 weeks (n = 5 per group) and 2 months (n = 6 per group) post-injection. Of note, the former groups were euthanized for biochemical and histological analysis without motor training. For Purkinje neuron calcium dysregulation during development, mice were given clozapine-Noxide (CNO) dissolved in 0.9% saline at 1 mg/kg or saline only, twice per day. Administration of CNO took place every day from P9–21 for preadolescent activation.

#### Stereotaxic virus injection and cannula implantation

In multi-fiber photometry experiments, we utilized a red fluorescent calcium sensor protein [35] to contrast the peri-centromeric GFP on the HSA21q-MAC [25] to independently record neural calcium activity by spectral separation. Stereotaxic surgeries were performed as previously described [30]. AAV vectors expressing hM3D-CTS DREADD were generated in AAV.CAG-FLEX vector. For ICM injection, P2-3 Pcp2-Cre pups received 4 μL AAV (1.0E10 GC) 2 mm posterior to lambda. For photometry, AAV1-CAG.Flex.NES-jRCaMP1a was injected into cerebellar nuclei (AP:-6.13, ML: ± 1.40, DV:-3.60 mm) and AAVrg.EF1a.Cre/AAV1Syn.NES.jRCaMP1a into ventrolateral thalamus (AP:-0.9, ML: ± 1.00, DV:-3.75 mm). Optical fibers (400μm, 0.48NA) were implanted above the injection sites.

### Histology & immunohistochemistry

Tissue processing and immunocytochemistry were performed exactly as described [37]. Mice were perfused with ice-cold PBS followed by 4% PFA. Brains were post-fixed for 24 h at 4 °C, cryoprotected in 30% sucrose, and sectioned at 40μm. Antibodies used: anti-Calbindin D-28K (CB300, Swant), anti-PCP4 (HPA005792; Millipore-Sigma), anti-VGluT2 (#135418, SYSY), anti-HSP25 (ADI-SPA-801-F), anti-VGAT (#131004, SYSY). Sections were imaged using Nikon Ti2 confocal microscope with 10x/0.45NA (lobe size), 20x/0.75NA (Hsp25 pattern), or 63x/1.40NA objectives (synaptic markers). Z-stacks were analyzed using IMARIS software (OXFORD Instrument).

### Western blotting

Cerebella were lysed in ice-cold RIPA buffer (150 mM NaCl, 0.1% SDS, 1% NP-40) supplemented with 2 mM NaF, 2 mM NaVO3, 2 mM PMSF, and 1× cOmplete protease inhibitor cocktail (Roche) for 10 min on ice. Insoluble debris was pelleted by centrifugation at 14,000 rpm for 10 min at 4 °C. Lysates were denatured in LDS sample loading buffer (Life Technologies) at 70 °C for 10 min, and electrophoresed on 10% Tris-Glycine gels (Bio-rad). Proteins were transferred to PVDF membranes and probed with primary antibodies for: DYRK1A (7D10; Abnova), RCAN1 (ab140131; Abcam), PDE9A (sc-376271; SantaCruz), Pericentrin (ab99341, Abcam), vinculin (Proteintech), and Actin (Sigma-Aldrich), and detected with HRP-conjugated secondary antibodies and ECL substrate (GE Healthcare). Band densitometry values were calculated using ImageJ software.

### Purkinje neuron sagittal stripe gene expression quantifications

Cerebella from TcMAC21 mice and littermates (n = 3 for P7/P14, n = 4 for adult) were analyzed for Hsp25 distribution. Sections (40μm, 200μm intervals) were evaluated within 2 mm square regions of lobular IX/X. Coexpression of Hsp25+/Calb+ and Hsp25-/Calb+ cells was quantified using ImageJ (Rasband, W.S., ImageJ, NIH, MD).

### In vivo two-region photometry recordings

#### Photometry setup

Excitation of the 560 nm (imaging) and 405 nm (isosbestic control) wavelengths were provided by a commercially available photometry system (Neurophotometrics, Model FP3002), which was controlled via the open-source software Bonsai [38]. Excitation light is directed on to a custom branching fiberoptic patchcord of three bundled 400 μm diameter 0.22NA fibers (BFP(3)_400/440/900–0.22_2m_FCM*−3xFC, Doric Lenses) by objective lens (Neurophotometrics, Model FP3002). RCaMP1a fluorescence from neurons below the fiber tip in the brain was transmitted via fiber optic patch cable back to the objective and was recorded. The multiple branch ends of the branching fiberoptic patchcord were connected to an array of fiberoptic rotary joints (FRJ_1×1_FC-FC, Doric Lenses) and coupled to two low autofluorescence patchcords (MFP_400/430/1100–0.57_1m_FC-ZF1.25_LAF, Doric Lenses) which is used to collect emission fluorescence from 1.25 mm diameter light weight ferrules (MFC_400/430–0.48_ZF1.25, Doric Lenses) using a mating sleeve (Doric SLEEVE_ZR_1.25). Bulk activity signals were collected using the PVCAM software, and data were further postprocessed and analyzed using custom MATLAB scripts.

#### Voluntary wheel running with photometry recordings

The mouse with photometry implants was head-fixed on the running wheel (diameter: 12 cm, width: 5 cm), which was housed in a dimly lighted, sound attenuated box. The wheel was fitted with a sensor to record rotations via a computerized monitoring system. Following four ten-minute habituation sessions (2/day) to the head-fixed conditions, wheel-running activity was monitored continuously for four consecutive days. The following parameters were recorded: Running velocity (m/min), Running bout frequency (number of discrete running episodes/day). A running bout was defined as any wheel rotation lasting ≥ 3 s, with intervals of > 10 s of inactivity denoting separate bouts. All measurements were conducted under standard laboratory conditions. Mice performed a voluntary Wheel Running task while we recorded bulk calcium signals from two regions, the cerebellar nuclei (CN) and motor thalamus (VL), simultaneously. We recorded at 30 Hz frequency with excitation alternating between 560 nm (calcium-dependent fluorescence) and 405 nm (calcium-independent fluorescence) excitation wavelengths, resulting in an effective frame rate of 15 Hz, sufficient for capturing jRCaMP1a fluorescence dynamics. Multi-Fiber Photometry Data Processing was performed as previously described [30].

### Quantification and statistical analysis

#### Behavior statistics reporting

Sample sizes were based on literature precedent, with randomized group assignment and blinded investigators. Five experimental cohorts included: Cohort 1 (n = 18 males, P45-60) for open field/ErasmusLadder; Cohort 2 (n = 24 pups of both sexes, P7-9) for USVs; Cohort 3 (n = 10 dams, P90-120) for retrieval; Cohort 4 (n = 11 of both sexes, P56-72) for photometry; Cohort 5 (n = 30 of both sexes) for chemogenetics, subdivided equally into hM3D-CTS + CNO, hM3D-CTS+vehicle, and RFP + CNO groups. Cohort 6 (n = 9 of both sexes) for rotarod test. Cohort 7 (n = 12 of both sexes, P45-60) for the open field test. All mice were behaviorally naïve. Data were analyzed using GraphPad Prism 10 with repeated measures ANOVA. Detailed statistics are reported in Table 1.

**Table 1:** Details of statistical tests and results.

Figure Panel	Figure title	Statistical test	Sample size (male/female)	Comparison	Test stat.	DF	P-value	
Fig. 1B	Missstep (session 1-4)	RM-ANOVA test; Sidak’s Post-Hoc test	Eu male: n = 9, TcMAC21 male: n = 9	Eu-TcMAC21, session1	F = 30.64	DFn = 1, DFd = 16	0.0124	*
				Eu-TcMAC21, session2			0.0124	*
				Eu-TcMAC21, session3			0.0010	**
				Eu-TcMAC21, session4			0.0009	***
Fig. 1C	Steptime (session 1-4)	RM-ANOVA test; Sidak’s Post-Hoc test	Eu male: n = 9, TcMAC21 male: n = 9	Eu-TcMAC21, session1	F = 5.867	DFn = 1, DFd = 16	0.6556	ns
				Eu-TcMAC21, session2			0.4677	ns
				Eu-TcMAC21, session3			0.0451	*
				Eu-TcMAC21, session4			0.0654	ns
Fig. 1D	Percentage longstep (session 1-4)	2way ANOVA-RM	Eu male: n = 9, TcMAC21 male: n = 9	Eu-TcMAC21, session1	F = 14.60	DFn = 1, DFd = 16	0.7732	ns
				Eu-TcMAC21, session2			<0.0001	****
Fig. 1E	Steptime (session 5-6)	2way ANOVA-RM; Sidak’s Post-Hoc test	Eu male: n = 9,TcMAC21 male: n = 9	Eu-TcMAC21, session5 post-rise	F = 92.11	DFn = 1, DFd = 16	<0.0001	****
				Eu-TcMAC21, session6 post-rise			0.0051	**
				Eu-TcMAC21, session5 pre-rise	F = 6.383	DFn = 1, DFd = 16	0.2662	ns
				Eu-TcMAC21, session6 pre-rise			0.2034	ns
Fig. 1G	Call amount	Unpaired t-test	Eu pups: n = 12 (5/7), TcMAC21 pups: n = 12 (6/6)	Eu vs. TcMAC21	F = 4.667	DFn = 11, DFd = 11	0.0168	*
	USV tonality	Unpaired t-test		Eu vs. TcMAC21	F = 1.677	DFn = 11, DFd = 11	0.0055	**
	USV frequency	Unpaired t-test		Eu vs. TcMAC21	F = 3.066	DFn = 11, DFd = 11	0.3514	ns
	Call duration	Unpaired t-test		Eu vs. TcMAC21	F = 1.895	DFn = 11, DFd = 11	0.1485	ns
Fig. 2B	Cross-sectional area, total cerebellum	Unpaired t-test	Eu: n = 8 (4/4), TcMAC21: n = 8 (4/4)	Eu vs. TcMAC21, molecular layer	F = 2.047	DFn = 7, DFd = 7	<0.0001	****
		Unpaired t-test		Eu vs. TcMAC21, Granule cell layer	F = 1.411	DFn = 7, DFd = 7	0.0002	***
				Eu-TcMAC21, Anterior Zone	F = 7.585	DFn = 1, DFd = 28	0.0400	*
	Cross-sectional area, molecular layer	Ordinary ANOVA; Sidak’s Post-Hoc test	Eu: n = 8 (5/3), TcMAC21: n = 6 (4/2)	Eu-TcMAC21, Central Zone	F = 8.515	DFn = 1, DFd = 12	0.6710	ns
				Eu-TcMAC21, Posterior Zone			0.6441	ns
				Eu-TcMAC21, Nadular Zone			0.2354	ns
	Cross-sectional area, granule cell layer	Ordinary ANOVA; Sidak’s Post-Hoc test	Eu: n = 8 (5/3), TcMAC21: n = 6 (4/2)	Eu-TcMAC21, Anterior Zone	F = 7.585	DFn = 1, DFd = 12	0.0386	*
				Eu-TcMAC21, Central Zone			0.4235	ns
				Eu-TcMAC21, Posterior Zone			0.8584	ns
				Eu-TcMAC21, Nadular Zone			0.4185	ns
Fig. 2E	VGluT2 puncta size	Unpaired t test	Eu: n = 14 (10/4), TcMAC21: n = 9 (5/4)	Eu vs.TcMAC21	F = 1.066	DFn = 8, DFd = 13	0.0006	***
	VGluT2-ir puncta intensity	Unpaired t test		Eu vs. TcMAC21	F = 1.761	DFn = 716, DFd = 998	<0.0001	****
Fig. 2F	VGAT puncta size	Unpaired t test	Eu: n = 9 (5/4), TcMAC21: n = 9 (5/4)	Eu vs. TcMAC21	F = 4.395	DFn = 8, DFd = 8	0.0495	*
	VGAT-ir puncta intensity	Unpaired t test		Eu vs. TcMAC21	F = 3.674	DFn = 651, DFd = 1029	<0.0001	****
Fig. 3D	VGluT2 puncta size	ANOVA test; Dunnett’s Post-Hoc test	*Pcp2::*hM3D-CTS(CNO): n = 5 (3/2), *Pcp2::*hM3D-CTS(veh) n = 5 (3/2), *Pcp2::*RFP(CNO) n = 4 (2/2)	*Pcp2::*RFP (CNO) vs. *Pcp2::*hM3D-CTS (CNO) vs. *Pcp2::*hM3D-CTS (veh)	F = 8.317	DFn = 2, DFd = 11	0.0296	*
	VGluT2-ir puncta intensity	ANOVA test; Tukey’s Post-Hoc test	*Pcp2::*hM3D-CTS(CNO): n = 5 (3/2), *Pcp2::*hM3D-CTS(veh) n = 5 (3/2), *Pcp2::*RFP(CNO) n = 4 (2/2)	*Pcp::*RFP(CNO) vs. *Pcp2::*hM3D-CTS(veh) *Pcp::*RFP(CNO) vs. *Pcp2::*hM3D-CTS(CNO) *Pcp2::*hM3D-CTS (CNO) vs. Pcp2::hM3D-CTS (veh)	F = 1588	DFn = 2, DFd = 7518	0.5897 <0.0001 <0.0001	ns **** ****
Fig. 3E	Percentage longstep	RM-ANOVA test; Dunnett’s Post-Hoc test	*Pcp2::*hM3D-CTS(CNO): n = 10 (6/4),	*Pcp2::*RFP (CNO) vs. Pcp2::hM3D-CTS (CNO), Day1	F = 8.863	DFn = 2, DFd = 27	0.9119	ns
			*Pcp2::*hM3D-CTS(veh) n = 10 (5/5),	*Pcp2::*RFP (CNO) vs. Pcp2::hM3D-CTS (veh), Day1			0.5594	ns
			*Pcp2::*RFP(CNO) n = 10 (6/4)	*Pcp2::*RFP (CNO) vs. Pcp2::hM3D-CTS (CNO), Day4			0.0002	***
				*Pcp2::*RFP (CNO) vs. Pcp2::hM3D-CTS (veh), Day4			0.1835	ns
Fig. 4F	Density of Hsp+ cells	Unpaired t test	Eu: n = 4 (2/2), TcMAC21: n = 4 (2/2)	Eu vs. TcMAC21	F = 1.176	DFn = 3, DFd = 3	0.013	*
Fig. 4G	Percentage of Hsp25+ cells	Unpaired t test	Eu: n = 4 (2/2), TcMAC21: n = 4 (2/2)	Eu vs. TcMAC21	F = 5.324	DFn = 3, DFd = 3	0.0012	**
Fig. 5D	Area under curve at z-scored dF/F of CN	2way RM-ANOVA ; Sidak’s Post-Hoc test	Eu: n = 5 (2/3), TcMAC21: n = 6 (3/3)	Eu vs. TcMAC21	F = 1.851	DFn = 1, DFd = 9	0.1968	ns
	Area under curve at z-scored dF/F of VL						0.7108	ns
Fig. 5E	Peak dF/F (CN, z-scored), for euploid mice	2way RM-ANOVA ; Sidak’s Post-Hoc test	Eu: n = 5 (2/3), TcMAC21: n = 6 (3/3)	Stationary vs. Running	F = 68.27	DFn = 1, DFd = 18	<0.0001	****
	Peak dF/F (CN, z-scored), for TcMAC21 mice		TcMAC21: n = 6 (3/3)	Stationary vs. Running			0.2283	ns
	Peak dF/F (VL, z-scored), for euploid mice		Eu: n = 5 (2/3)	Stationary vs. Running			0.0001	***
	Peak dF/F (VL, z-scored), for TcMAC21 mice		TcMAC21: n = 6 (3/3)	Stationary vs. Running			0.0369	*
Fig. 5G	Pearson’s correlation coefficients	Paired t test	Eu: n = 5 (2/3)	Stationary vs. Running	F = 6.406	DFn = 1, DFd = 78	0.0197	*
		Paired t test	TcMAC21: n = 6 (3/3)	Stationary vs. Running	F = 16.91	DFn = 1, DFd = 42	0.0076	**
Fig. 5H	Discrimination Index	Unpaired t test	Eu: n = 5 (2/3), TcMAC21: n = 6 (3/3)	Eu vs. TcMAC21	F = 1.804	DFn = 4, DFd = 5	0.0007	***

#### Multi-fiber photometry data analysis

Task phase activity was quantified as the area under the curve (AUC) of z-scored ΔF/F responses using MATLAB trapz function. To facilitate comparison across mice, ΔF/F responses were zscored and shifted above 0. For regional correlations, Pearson’s correlation coefficients were calculated between brain regions. To control for long photometry responses, timeseries were circularly permuted (15-25 frame offset) during running bouts. State discrimination was quantified using the following discrimination index (*DI*):

*DI* = |mean (*correlation_running*) – mean (*correlation_stationary*)| /(std(*correlation_running*) + std(*correlation_stationary*)); *correlation_running*: Pearson’s r between cerebellar nuclei and motor thalamus during running epochs; *correlation_stationary*: Pearson’s r during stationary epochs.

## Results

### Impaired motor control and altered vocalizations in TcMAC21 mice

**Fig. 1:**
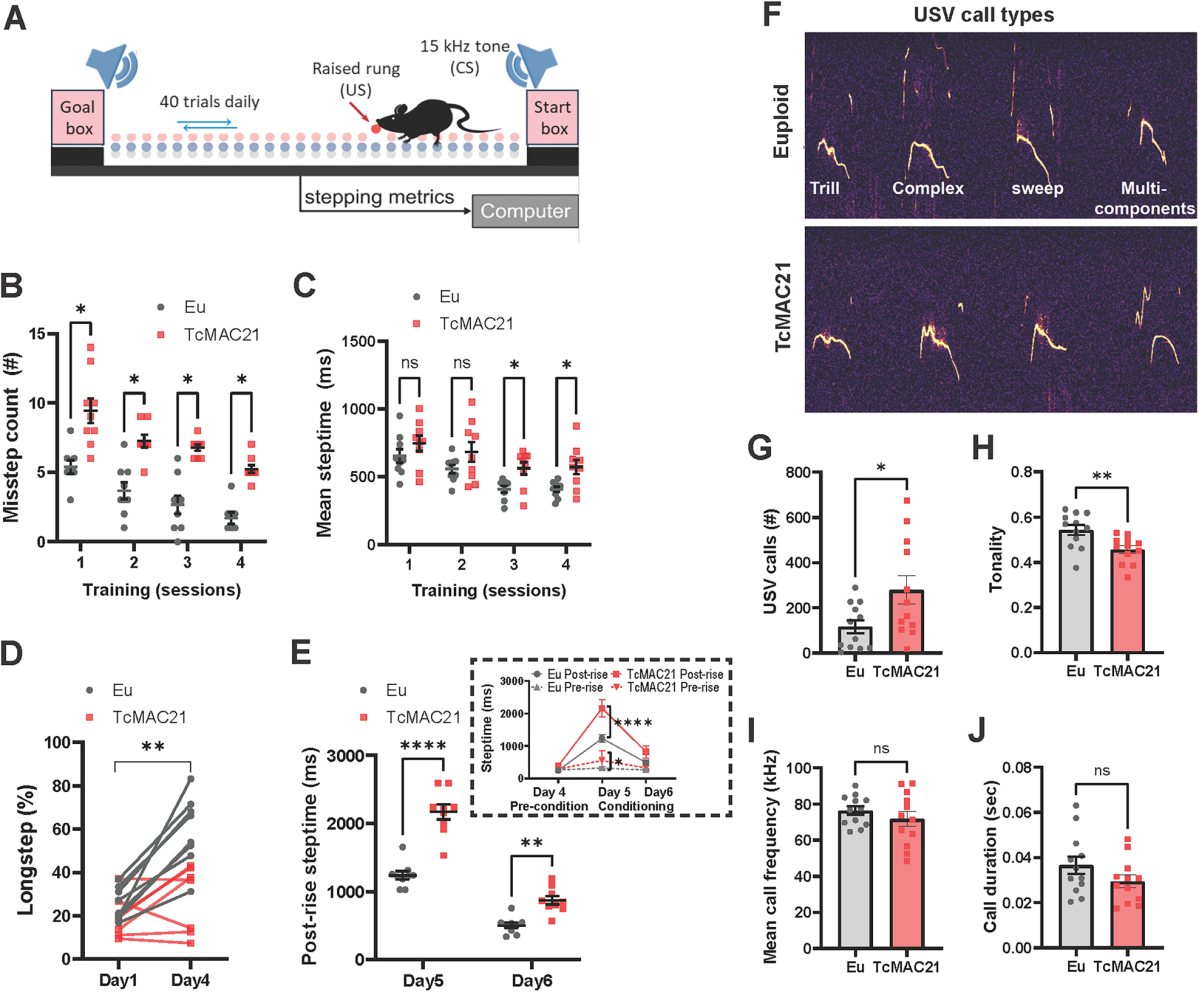
TcMAC21 mice exhibit lower motor control skill and altered vocalization. **A** Schematic presentation of the Erasmus Ladder which consists of a horizontal ladder situated between two shelter boxes. The mouse begins the task from inside the start box for a random time interval that varies between 9 and 18 s before it is allowed to walk on the ladder. When the time interval has passed, the LED light in the start box turns on and the mouse is allowed to start. The light remains on until the mouse reaches the goal box. Inter-limb coordination is tested during Days 1-4. Each daily session consisted of 40 trials, during which the mice had to walk back and forth between the goal and start boxes. Mice usually stepped on the upper rungs and only infrequently touched the lower ones, referred to as missteps. Locomotion adaptation is tested during challenge sessions (Days 5-8) when the mice learned to adapt their walking patterns in response to a 15 kHz auditory stimulus (CS) preceding the appearance of a raised rung (US) in their pathway. The US was located on the right side of the mouse and its movement specifically depended on the predicted position of the mouse on the ladder but was otherwise randomized. The blue and gray dots represent the upper and lower rungs, respectively. The position of the obstacle is indicated with a red dot and arrow during the challenge sessions. **B** Mean number of missteps per trial during the inter-limb coordination test were obtained from n = 9 mice of each genotype. TcMAC21 mice made significantly more missteps when traversing the ladder rungs compared to euploid controls. **C** TcMAC21 steptime had a longer latency during inter-limb coordination test. **D** TcMAC21 mice made a lesser proportion adapting long-stride pattern. **E** The first day of the challenge session (Day 5), TcMAC21 exhibited a greater increase in mean latency (“post-rise step time”) in response to the raised rung. **F** Representative sonograms of the four types of neonatal separation vocalizations. **G** TcMAC21 neonates (n = 12 neonates of each genotype) made significantly more total number of calls and **H** reduced vocalization tonality (signal-to-noise ratio) during separation-induced vocalizations. **I** No genotype difference in the mean vocalization frequency or **(J)** duration of calls produced. Data presented on a mouse-by-mouse basis. Data represent mean ± SEM.; *p < 0.05; **p < 0.01; black: Eu, red: TcMAC21.

To determine whether humanized trisomic mice (TcMAC21) exhibit deficits in complex, multi-joint motor behaviors similar to individuals with DS [1, 39–41], we assessed locomotor deficits in TcMAC21 mice using the ErasmusLadder task [28, 42, 43], which evaluates inter-limb coordination and cerebellar learning (Fig. 1A) while minimizing physical confounds [44].

Before motor testing, we evaluated changes in limb development in young male mice (n = 10/strain, femur length (mm): Eu 13.87 ± 0.37, TcMAC21 13.57 ± 0.65; tibia length (mm): Eu 18.07 ± 0.42, TcMAC21 18.04 ± 0.69, p = 0.079, Unpaired t-test; Supplementary Figure 1A, B). TcMAC21 mice could not be discriminated from control littermates in these assessments. This is particularly important because, in comparison to their male counterparts, female TcMAC21 mice exhibit significantly greater weight deficits (n = 10 females/strain; body weight in 9–10-week-old female TcMAC12 mice: 15.50 ± 1.947 g vs. Euploid mice 23.70 ± 2.111 g, mean ± SEM.; ***p < 0.001, Unpaired t-test; Supplementary Figure 1C) [45], therefore we restricted ErasmusLadder testing to males to isolate cerebellar circuit dysfunction from confounding weight-related effects.

During unperturbed training sessions, TcMAC21 (n = 9) mice exhibited motor impairments compared to euploid (n = 9) littermates; making more missteps (Fig. 1B; 2-way ANOVA with repeated measures, trisomy effect F_(1, 12)_ = 30.64, p < 0.001; session effect F_(3, 32)_ = 0.5022, p < 0.001) and showing extended response times (Fig. 1C; 2-way ANOVA with repeated measures, trisomy effect F_(1, 16)_ = 5.867, p = 0.027; session effect F_(2.319, 37.10)_ = 22.75, p < 0.0001). By day 4, euploid mice adopted long-stride patterns to reduce steps between goals, while TcMAC21 mice did not (Fig. 1D; 2-way ANOVA with repeated measures, trisomy effect F_(1, 13)_ = 12.05, p = 0.004, session effect F_(1, 13)_ = 30.01, p = 0.0001).

In challenge sessions (days 4-8), mice encountered obstacle rungs (US) preceded by warning tones (CS) with 250-ms intervals. This paradigm tests climbing fiber-dependent conditional motor learning [46, 47]. TcMAC21 mice showed impaired learning, failing to avoid obstacles following tone cues (Fig. 1E, individual data points were graphed and summarized data displayed in the inset; 2-way ANOVA with repeated measures and Sidak’s Post-Hoc test, session5 post-rise p < 0.0001, session5 pre-rise p = 0.0351), indicating deficits in associative motor adaptation.

To confirm that motor coordination deficits generalize across both sexes, we also assessed TcMAC21 mice using the accelerated rotarod test, which revealed consistent impairments in both male (n = 5/strain) and female (n = 4/strain) mice (Supplementary Figure 2A, p < 0.0001 for TcMAC21 vs. euploid, 2-way ANOVA with repeated measures; in subsequent comparison within TcMAC21 strain p = 0.4795 for males vs. females, not significant). Furthermore, basic motor and motivational measures showed no differences between sexes or strains (Open field maze, Supplementary Figure 1D–F, n = 6-12 mice per strain both sexes, total distance traveled (male/female): p = 0.217/p = 0.239, time spent in center(male/female): p = 0.863/p = 0.363, time spent in margin(male/female): p = 0.836/p = 0.558, Unpaired t-test), indicating that HSA21 effects were specific to motor learning and adaptation rather than general motor or motivational capabilities.

Cerebellar dysfunction often affects vocalization across neurological conditions [48]. Mouse models with cerebellar circuit mutations and other DS preclinical models [49–51] consistently exhibit vocalization deficits, pointing to a potentially shared neural pathway underlying vocalizations and motor control. Analysis of P8 pup isolation calls revealed that all mice produced separation calls during isolation (n = 12 mice per strain, example spectrograph of these calls shown in Fig. 1F). Even though the distribution of USV call numbers in TcMAC21 pups showed some overlap with euploid littermates, statistical analysis confirmed a statistically significant increase in call production in TcMAC21 pups. Tonality analysis of isolation calls showed significantly less frequency modulation in TcMAC21 calls (Fig. 1G, H; Number of calls t_(22)_ = 2.347, p = 0.0283, Tonality of USVs t_(22)_ = 3.078, p = 0.0055, unpaired t-test), while call duration and mean frequency remained unchanged (Fig. 1I, J; Mean call frequency t_(22)_ = 0.9520, p = 0.3514, Call duration t_(22)_ = 1.497, p = 0.1485, unpaired t-test). This finding is consistent with previous studies indicating that cerebellar dysfunction can enhance vocalization output and the heterogeneity within the TcMAC21 group is consistent with variable expressivity often observed in complex genetic disorders.

Next, we tested whether these changes in vocalizations altered maternal responses in the maternal retrieval tests. Increased USVs from TcMAC21 pups were paired with shorter retrieval latencies (Supplementary Figure 1G, H; 2-way ANOVA with mixed-effects, trisomy effect F_(1, 27)_ = 2.661, p = 0.0045). However, TcMAC21 dams showed longer latencies retrieving euploid pups (Supplementary Figure 1I; 2-way ANOVA with mixed-effects, trisomy effect F_(1, 27)_ = 6.826, p = 0.0145), indicating bidirectional disruption of social communication where TcMAC21 pups enhance vocalization while TcMAC21 dams show reduced maternal responsiveness. A control experiment using a two-way shuttle box demonstrated that female TcMAC21 mice successfully performed auditory cue-based avoidance behavior, confirming normal hearing and auditory processing capabilities in TcMAC21 animals (Supplementary Figure 1J). This finding indicates that the longer retrieval latencies exhibited by TcMAC21 dams when responding to pup vocalizations reflect specific deficits in maternal responsiveness to social communication cues rather than underlying auditory impairments.

### Trisomic cerebellar vermis sizes are disproportionately reduced and climbing fiber synapses are enlarged

**Fig. 2:**
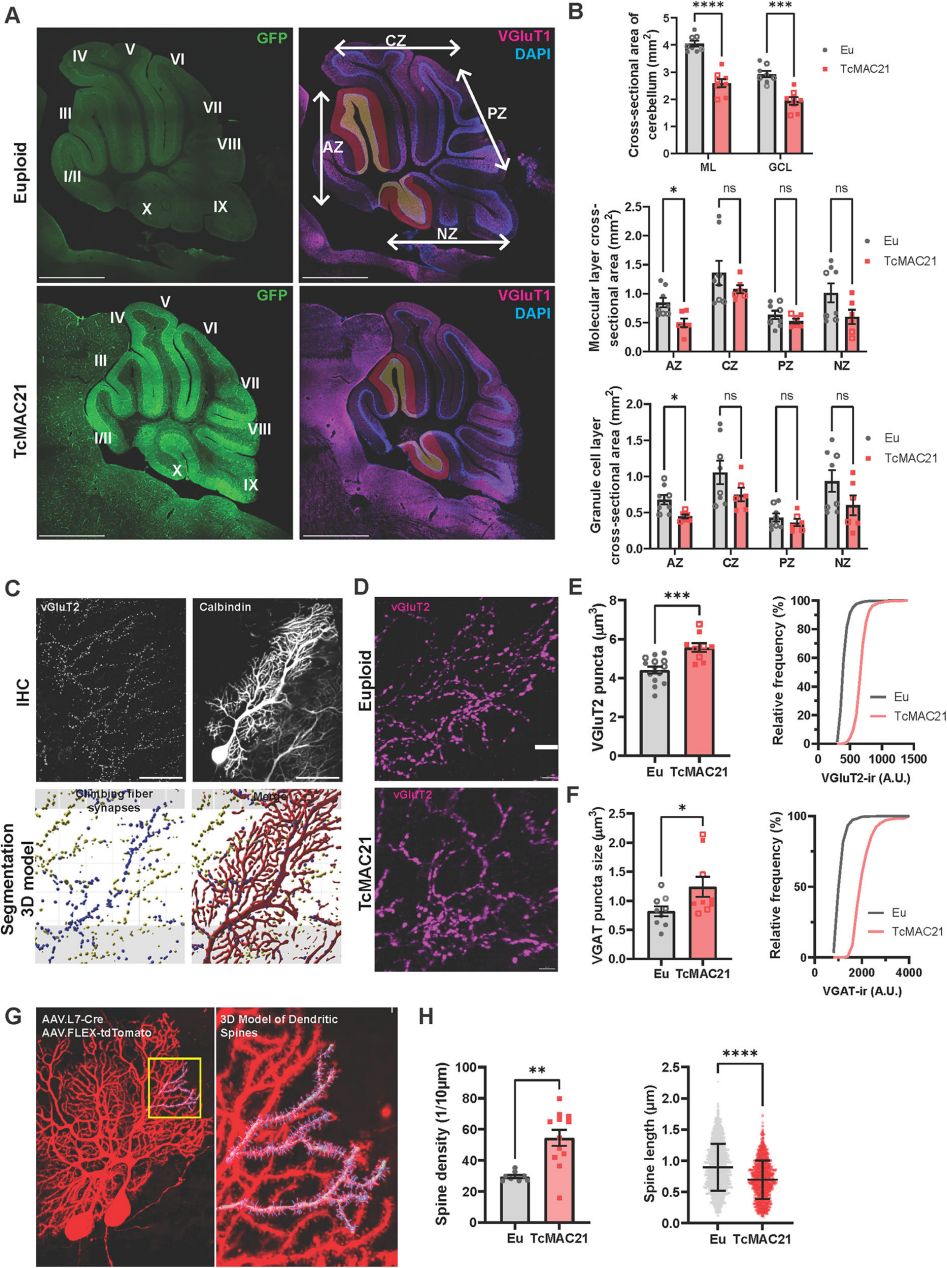
TcMAC21 cerebella feature disproportionally reduced anterior and nodular zones, altered pre- and postsynaptic morphology. **A** Representative euploid (top) and trisomy (bottom) midsagittal cerebellar sections. The green fluorescence represents GFP expression from the artificial human chromosome 21 present in TcMAC21 mice but absent in euploid controls; all other labeling represents specific antibody immunoreactivity (VGluT1, magenta; DAPI counterstain, blue). Fissures separate the vermis into lobes (left panels, designated by roman numerals). Anterior vermis (AZ) located anterior to the primary fissure, central vermis (CZ) located between the primary fissure and the horizontal fissure, and nodular vermis (NZ) included the nodulus, which is separated from the posterior vermis (PZ) by the posterolateral fissure (right panels). **B** The analysis of cerebellum morphology. Total cross- sectional area (top) of the molecular layer (ML) and granule cell layer (GCL) measured in the cerebella of TcMAC21 (n = 8) and euploid (n = 8) mice. Each data point represents one mouse, with solid symbols indicating males and open symbols indicating females. Data from the AZ, CZ, PZ, and NZ were discretely represented by (middle) the ML size or (bottom) the GCL size showed a significant decrease in AZ in TcMAC21 cerebella. **C** Confocal image showing an example of a Purkinje neuron transfected with AAV expressing tdTomato and double stained with vGluT2 (top panels); a high magnification view of a three-dimensional reconstruction of presynaptic vesicle clusters and dendritic shafts from the same neuron (bottom). Scale bar: 50 μm. **D** Example of a stretch of synaptic vesicle clusters of climbing fiber synapses, labelled using VGluT2 antibody staining (magenta). Scale bar: 10 μm. Quantifications of **E** VGluT2 staining puncta size and intensity distribution, and **F** VGAT staining puncta size and intensity distribution were obtained from 9 – 14 mice of each genotype. Both showed a significant trisomy effect of size enlargement. Each data point represents a mouse, and solid data points represent males; open data points represent females. Bars represent mean ± SEM. ***p < 0.001 by t test. **G** A Confocal image showing an example of two Purkinje neurons transfected with AAV.L7- Cre/AAV.FLEX-tdTomato (red) (left), and a high magnification view of a dendritic segment from the neuron on the right with a three-dimensional reconstruction of spine heads and shafts (right). **H** Quantifications were obtained from n = 4 mice of each genotype, showing trisomy effects on an increased spine density and a decreased spine shaft length in TcMAC21 Purkinje neurons. 2-3 representative fields of view were measured per mouse; each data point represents a field of view (left panel). Tukey boxplots represent median, the minimum and maximum values (right panel).

A previous study confirmed smaller cerebellar size in TcMAC21 mice [25], but our systematic assessment of TcMAC21 cerebellar lobules revealed disproportional hypoplasia in specific regions (Fig. 2A and B, n = 8 mice per strain (four males and four females per group); Molecular layer (ML) cross-section t_(14)_ = 7.821, p < 0.0001, Granule cell layer (GCL) cross-section t_(14)_ = 5.569, p = 0.0001, unpaired t-test and each data point represents one mouse). The anterior (I-III) and nodular lobes (IX/X) showed selective decreases in ML and GCL (Fig. 2B; 2-way ANOVA with repeated measures at anterior lobe (AZ) ML p = 0.037, GCL p = 0.038, unpaired t-test, and each data point represents one mouse), indicating differential effects of HSA21 triplication across lobules.

Purkinje neurons in the cerebellar cortex receive two types of glutamatergic inputs, the climbing fibers and the parallel fibers, and GABAergic inputs from molecular layer interneurons. We performed high-resolution confocal analysis of climbing fiber synapses and revealed enlarged VGluT2-positive terminals in TcMAC21 cerebella (Fig. 2C). Climbing fiber afferents, originating from inferior olive, are essential for cerebellar learning, as there is broad agreement that somatosensory feedback drives plasticity in Purkinje neurons [52–56]. VGluT2-positive synapses extended to around 80% of the molecular layer height (Supplementary Figure 4A–C). We observed increased puncta size of trisomic VGluT2-positive synapses (Fig. 2D, E, n = 7-8 mice per strain with both males and females represented; Puncta size t_(21)_ = 4.041, p = 0.0006, unpaired t-test and each data point represents one mouse). VGluT2 immunoreactivity intensity was also increased, suggesting altered presynaptic vesicle content.

Furthermore, analysis of inhibitory synaptic inputs to Purkinje neurons showed increased inhibitory synapse size with decreased presynaptic vesicle pool in TcMAC21 mice (Fig. 2F, n = 7-8 mice per strain with both males and females represented; Puncta size t_(664)_ = 4.410, p < 0.0001, unpaired t-test, and each data point represents one mouse). While molecular layer interneurons coordinate to provide negative feedback to control Purkinje neuron firing [57, 58], there is also evidence for GABAergic deficits in individuals with DS [59, 60]. Additionally, analysis of both male and female TcMAC21 mice confirmed that cerebellar morphological changes and climbing fiber synapse alterations were consistent across sexes (n = 3-4 mice per strain in each sex group; Supplementary Figure 2B, C). These changes, combined with observed gait abnormalities [61], indicate disrupted climbing fiber-Purkinje neuron connectivity as a key pathogenic mechanism [62].

### Calcium homeostasis in developing Purkinje neurons regulates adult cerebellar afferent synapses

Next, we examined whether trisomy of HSA21 affects the spine morphology of Purkinje neurons. To sparsely label Purkinje neurons and their dendritic spines, we systematically delivered Purkinje neuron-specific minimal promoter (0.8-kb) [63] driven CRE virus (AAV.L7-6.Cre PHP.eB Serotype, 1 × 10^10^ VG per animal) and conditional tdTomato expression vector via intracisterna magna (ICM) injection (Supplementary Figure 4D–F). We observed increased spine density but decreased spine length in TcMAC21 mice at P45 (Fig. 2G and H, 2–3 representative fields of view were measured per mouse, with n = 4 animals per strain; Spine density t_(31)_ = 3.455, p = 0.0016, Spine length t_(3427)_ = 16.80, p < 0.0001, Unpaired t-test and each data point represents a field of view), suggesting a shift toward immature, filopodia-like spines [64] less capable of supporting stable synaptic connections and plasticity.

**Fig. 3:**
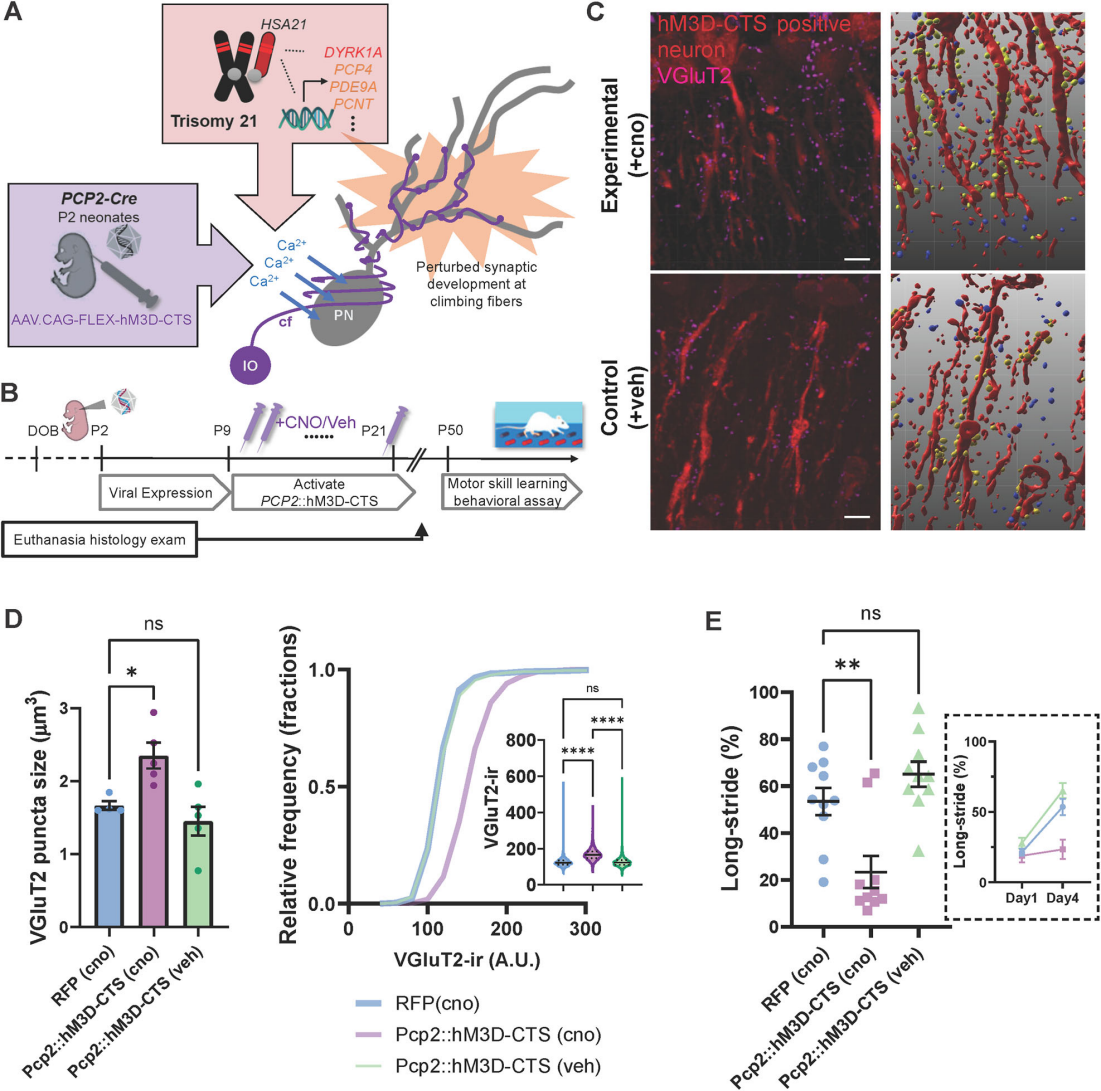
Developing Purkinje neuron calcium imbalance induces a persistent morphological change in cerebellar afferent synapses and a deficit in inter-limb adaptation task in adult. **A** Schematic to determine whether dysregulation of developing Purkinje neuron calcium balance leads to long-lasting changes in cerebellar circuit function. **B** Experimental timeline for developing Purkinje neuron calcium dysregulation. CNO was administered from P9 to P21 to mice expressing hM3D-CTS or RFP in Purkinje neurons, and behavioral testing was conducted 30 days later at P50; an independent cohort was established for confocal analysis of climbing fiber synapses to control for cerebellar plasticity effects induced by motor training. **C** Representative confocal images (left) of Purkinje neurons expressing hM3D-CTS (co-expressed RFP reporter, red) co-stained with VGluT2 (magenta), and a three-dimensional reconstruction (right) of virally transduced dendrites (Red), neighboring presynaptic terminals (Yellow) and non-adjacent terminals (Blue), from mice that were CNO-treated (top) or vehicle-treated (bottom). Scale bar: 10 μm. **D** Quantification of VGluT2 staining puncta size and intensity was obtained from n = 4 – 5 mice of each treatment group (both sexes included), showing a significant enlargement in climbing fiber afferent synapses in CNO-treated hM3D-CTS mice during the early postnatal period compared to controls. **E** CNO-treated hM3D-CTS mice showed impaired adaptation to the long-stride pattern. Individual data points of the last (Day4) training session were graphed and trend across days displayed in an inset. Data are presented as the mean ± SEM.; *p*(RFP vs. Pcp2::hM3D-CTS_veh_) = 0.3035, **p(RFP vs. Pcp2::hM3D-CTS_cno_) = 0.0027; Ordinary ANOVA with post-hoc Dunnett’s test.

One potential cause of the altered dendritic spine morphology is dysfunctional calcium buffering. Dendritic spines are the primary sites of synaptic input, and their size and shape are closely linked to synaptic strength and plasticity. Altered calcium buffering, resulting from impaired calcineurin activity, may lead to an increase in dendritic spine density, as seen in other models of synaptic dysfunction [65–67]. The overexpression of HSA21 genes like *DYRK1A*, *PDE9A*, *PCP4*, and *PCNT* in Down syndrome disrupts calcium homeostasis (Table 2), primarily by suppressing the calcineurin pathway [19, 68]. Transcriptomic analysis from TcMAC21 mice confirmed a 1.4- to 1.8-fold increase in mRNA levels of *Dyrk1a, Pde9a, Pcp4, Rcan1*, and *Pcnt*, consistent with the expected gene dosage effect from the additional HSA21 copy [25]. Western blot analyses from cerebellar lysates confirmed increased protein abundance of these gene products in TcMAC21 mice (Supplementary Figure 3A, B), supporting functional overexpression within cerebellar tissue. This disruption can result in elevated intracellular calcium levels due to impaired calcium buffering [69, 70]. Given that calcium signaling is crucial for synaptic development, plasticity, and function [71, 72], we hypothesize that this calcium dysregulation directly contributes to synaptic abnormalities in the cerebellar circuit. We expected that developmental perturbation of Purkinje neuron calcium homeostasis can have a variety of effects on cerebellar circuit formation, thus creating phenotype resemblance to enlarged climbing fiber synapses in TcMAC21.

**Table 2:** Gene triplication on human chromosome 21 controls intracellular calcium.

HSA21 gene	Effector	Mechanism of action	Reference
*DYRK1A*	GluN2A, CaMKIIδ, CEP97	Surface expression and channel activity of NMDA receptors, intracellular signaling molecules in the cytoplasm	PMID: 25368549 PMID: 26067684 PMID: 34787650
*PDE9A*	Phospholamban	elevating the cytoplasmic [Ca^2+^]	PMID: 28649129
*PCP4*	Calmodulin	mobilize [Ca^2+^] in context dependent manner	PMID: 10751438 PMID: 23204517
*PCNT*	Calmodulin,PCP2	Distribution of calcium-selective channel on primary cilia	PMID: 25031429 PMID: 15337773

To test this hypothesis, we examined the developmental relationship between calcium buffering capacity and synaptic morphology in Purkinje neurons in vivo. To investigate whether these developmental defects are cell-autonomous to Purkinje neurons, we applied the Purkinje neuron-specific *Pcp2-Cre* to drive targeted recombination in these neurons beginning at postnatal day 2 (P2). We then used chemogenetic activation of primary cilia, a transient signaling organelle capable of controlling circuit formation [73], by coupling mutant GPCRs (DREADDs) with Gq to activate phospholipase C, leading to increased intracellular calcium upon clozapinen-N-oxide (CNO) stimulation (Fig. 3A). The injection contained either AAV.CAG.FLEX-hM3D-CTS (*Pcp2::*hM3D-CTS group) or a control construct expressing tdTomato (RFP group). We induced hM3D-CTS at P9–P21 with CNO (1.0 mg/kg) or saline-only (veh), given orally twice daily [74], during cerebellar circuit refinement (Fig. 3B) [75, 76]. We found that DREADD-mediated increases in intracellular calcium levels in Purkinje neurons during postnatal developmental period have a long-lasting effect of enlarged climbing fiber synapses and increased VGluT2 immunoreactivity in their presynaptic terminals (Fig. 3C, D; sample sizes: *Pcp2::*hM3D-CTS + CNO (n = 5), *Pcp2::*hM3D-CTS + veh (n = 5), and RFP + CNO (n = 4), with both sexes represented in each group; Puncta size analysis, 2-way ANOVA with post-hoc Dunnett’s test *Pcp2::*hM3D-CTS (CNO) vs. RFP (CNO): adjusted p = 0.0006, *Pcp2::*hM3D-CTS (CNO) vs. *Pcp2::*hM3D-CTS (veh): adjusted p = 0.0006; puncta intensity analysis, 2-way ANOVA with Dunnett’s post-hoc test p < 0.0001). These findings suggest that the TcMAC21 mutants and manipulation of the intracellular calcium pathway converge on a common mechanism, leading to similar structural alterations at Purkinje neuron synapses, indicative of a shared disruption in synaptic architecture and development.

Since the adult cerebellar afferent synapse phenotype shows similarity between TcMAC21 mice and Pcp2::hM3D-CTS (CNO) mice, we next used inter-limb control adaptation behavior to determine the adult functional significance of the perturbed intracellular calcium pathway in developing Purkinje neurons. We assessed locomotion performance in *Pcp2::*hM3D-CTS mice using ErasmusLadder (n = 10 per group, with both sexes presented in each group) and found that training-induced step pattern change was suppressed in the CNO-treated experimental hM3D-CTS expressing group when compared to control groups (vehicle-treated, or RFP expressing) (Fig. 3E, in the last training day (Day4), RFP (CNO) vs. *Pcp2::*hM3D-CTS (CNO): p = 0.0002 and RFP (CNO) vs. *Pcp2::*hM3D-CTS (veh): p = 0.1835, 2-way ANOVA with repeated measures). Taken together, these results point to a long-term effect of intracellular calcium disturbance in neurons during neonatal development on climbing fiber synapses to cerebellar Purkinje neurons, with enlarged volume of the VGluT2 boutons accompanied by decreased locomotor performance.

### Trisomy of HSA21 alters the organization of Purkinje neuron sagittal stripe gene expression in developing and adult cerebellum

**Fig. 4:**
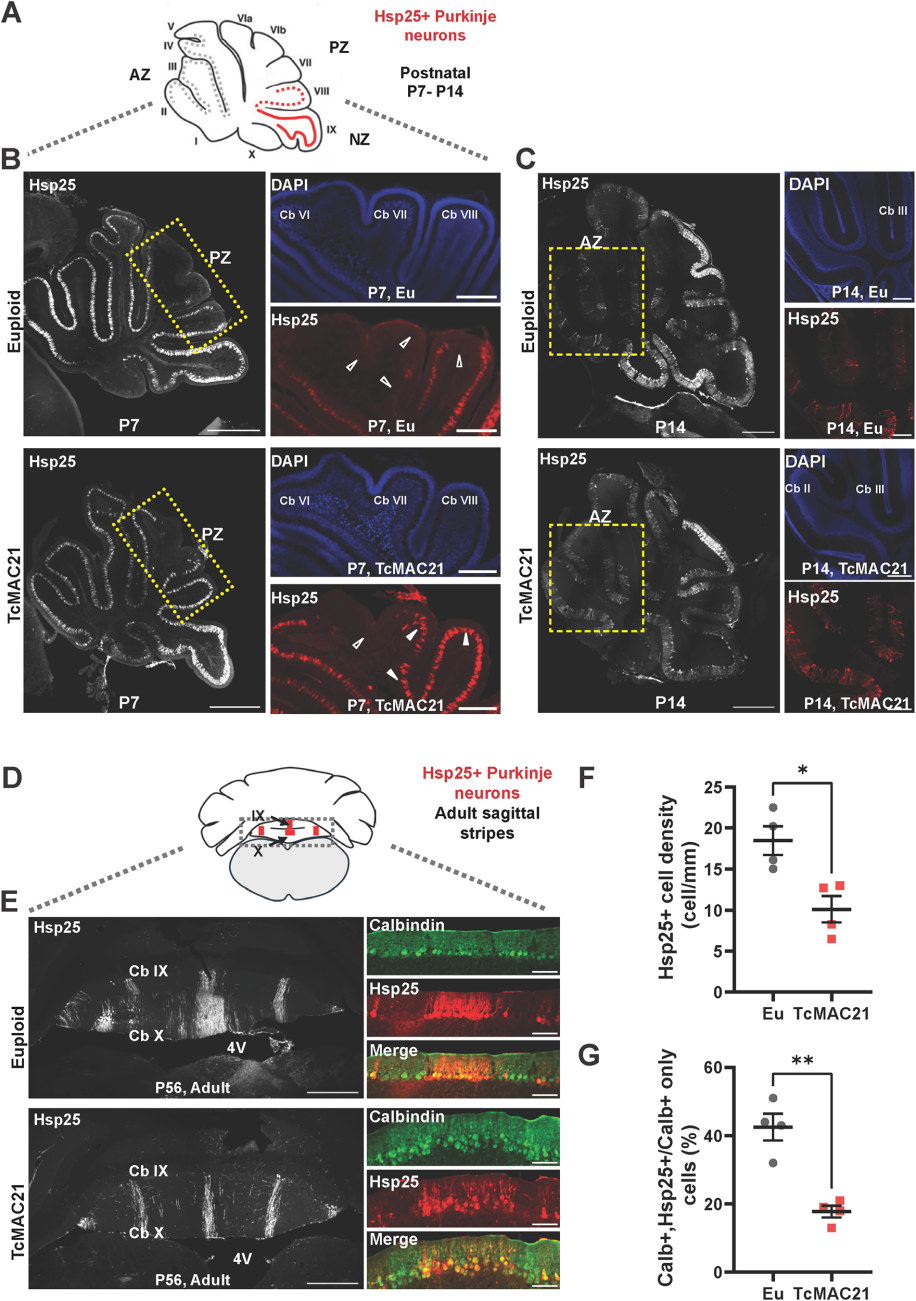
Developing and adult TcMAC21 cerebella exhibited disrupted Purkinje neuron subtype antigen Hsp25 expression pattern. **A** The sagittal schematics above the data panels indicate the lobules that show parasagittal stripes of Hsp25 (Red), or its absence (Gray); and correspond to the tissue sections shown in panels below. **B** At P7, Hsp25 immunoreactivity (ir)(Hsp25, gray or red) shows diminished expression in the posterior lobe (dotted rectangle) of euploid cerebella (*top*, empty arrowheads), while expression remains widespread in TcMAC21 mice (*bottom*, filled- arrowheads), indicating disrupted patterning. The presence of DAPI counterstaining (blue) indicated the absence Hsp25-ir was not due to artifact. Scale bars: 500 μm on left-panels and 100 μm on the right-panels **C** The atypical HSP25 distribution persists at P14 in TcMAC21 cerebella (*bottom*), differing from the typical restricted pattern seen in euploids (*top*). Note the expanded expression domain (dashed rectangle) compared to the normal restricted pattern. **D** In adult mice, the coronal schematics indicate Hsp25-positive Purkinje neurons show specific patterning in the nodular zone of control cerebella. **E** TcMAC21 mice display reduced Hsp25-positive Purkinje neurons (Hsp25, gray or red) in the medial parasagittal stripes, but wider parasagittal stripes were seen in euploid mice. Scale bars: 500 μm and 50 μm (insets). Quantification was obtained from n = 4 mice of each genotype (both sexes included), showing a significantly decrease in both the cell number **F** and percentage **(G)** of Hsp25+ Purkinje neurons in adult TcMAC21 mice.

The lobule-specific effects (Fig. 2A, B) observed may reflect the differential vulnerability of cerebellar regions to HSA21 gene dosage, as the AZ matures earlier in development and expresses neurogenesis genes like *EURL/C21ORF91* that could prematurely terminate progenitor cell proliferation [77, 78]. Alternatively, altered Purkinje neuron differentiation and patterning could disrupt Sonic Hedgehog signaling to granule cell precursors and consequently affect lobule formation in a spatially-specific manner [79, 80]. In TcMAC21 mice, the nodular zone, featuring strong expression of Hsp25 and tyrosine hydroxylase parasagittal stripes (Supplementary Figure 4G) [37, 81], is significantly reduced in size, along with the anterior zone (Fig. 2A, B). This reduction prompted a detailed analysis of the distribution and abundance of Hsp25-positive Purkinje neuron subtypes. The majority of Purkinje neurons across the vermis and hemispheres express Hsp25 in the first week after birth [82], but the expression pattern in the anterior lobe is transient and diminishes over time. Indeed, we observed that Hsp25 immunoreactivity was already diminishing at the posterior lobe of the euploid cerebella, but that the expression remained widespread in TcMAC21 (Fig. 4A, B), indicating disrupted patterning. This atypical organization persists into the second postnatal week (P14), potentially impeding circuit formation and refinement, contrary to the typical restricted distribution of Hsp25-positive (Hsp25+) Purkinje neurons (Fig. 4C).

We then proceeded to examine whether there were differences in the adult zonal patterning. To determine the abundance of Purkinje neuron subtypes, we measured the number of HSP25+ cells in the middle parasagittal zone in the lobules IX and X because of their consistent topographical patterning in the adult (Fig. 4D, E) [37, 83]. In adult TcMAC21, we found that Hsp25+ cells were less abundant and reduced percentages of Hsp25+ neurons were reproducibly found in TcMAC21 mice (Fig. 4F and G, n = 4 mice per strain with both sexes represented in each group; Cell number t_(6)_ = 3.491, p = 0.0130, Cell percentage t_(6)_ = 5.784 p = 0.0012, Unpaired t-test), suggesting selective loss of the Hsp25+ cell type in trisomic mice. These findings demonstrate that trisomy of HSA21 disrupts the typical zonal patterning of Purkinje neurons during early postnatal development, with abnormal persistence of Hsp25 expression in TcMAC21 mice, leading to altered cerebellar organization that may impede proper circuit formation and refinement into adulthood.

### Altered cerebello-thalamic responses during locomotion in TcMAC21 mice

**Fig. 5:**
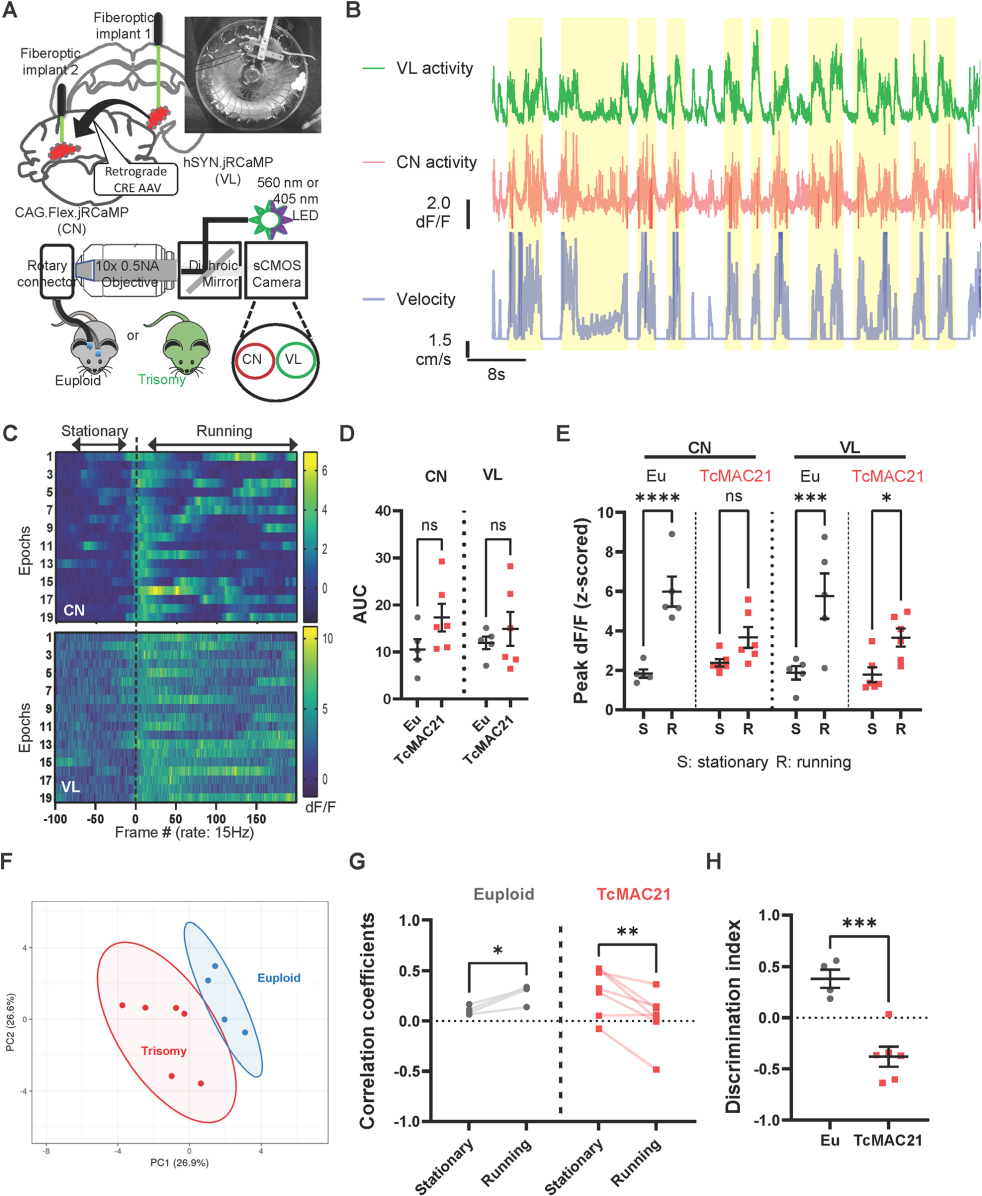
Trisomy 21 alters the temporal pattern of the cerebellothalamic activation during locomotor activity. **A** Schematic setup. An optic bundle consists of two optical fibers that connect the optical cannula implanted at the interposed nucleus (IPN) and ventrolateral nucleus of thalamus (VL) to the focal plane of the objective. A dichroic mirror separates excitation and detection pathways and selects the detected wavelength range with an emission filter. A complementary metal-oxide semiconductor (CMOS) camera sensor creates an image of the two-region fiber array. We used a 560-nm LED to excite RCaMP. Upper right: example video camera image of voluntary wheel running with simultaneous two-region fiber photometry recording. **B** Example trial recordings. Top to bottom: bulk RCaMP ΔF/F (VL), ΔF/F (CN), and velocity traces recorded simultaneously across two regions from an euploid mouse. Shaded area: running epochs. **C** Example peristimulus time histogram (PSTH) responses from two brain regions of an euploid mouse. Top: CN recordings, bottom: VL recordings. Heatmap colors in each row indicate ΔF/F amplitude of one running epoch. Dashed lines indicate running onset. **D** Average activity (area under z-scored responses) for euploid (n = 5) vs TcMAC21 (n = 6) during 10-min recordings. No significant differences, ANOVA followed by Sidak’s test. Data are mean ± SEM. **E** Locomotor state-dependent changes in mean response magnitude (maximum Z scored response averaged across behavior epochs). Individual mice shown, including mean ± SEM. *p < 0.05, ***p < 0.001, ****p < 0.0001 by two-way RM-ANOVA with Sidak’s multiple comparisons. **F** Multidimensional analysis using the eight temporal and amplitude bulk activity (z-scored ΔF/F) measurements from interposed nucleus and motor thalamus of TcMAC21 and euploid mice during locomotor state transitions. Each data point represents one mouse, mapped in principal component space. Neuronal responses during locomotor state transitions clustered according to genotype. **G** Pairwise Pearson’s correlations of bulk activity between motor thalamus and cerebellar nuclei recordings during each locomotor epoch for euploid (n = 5) and TcMAC21 (n = 6) mice. Data points represent correlation mean across epochs per mouse, including mean ± SEM. Euploids showed significantly increased correlation, while TcMAC21 mice showed significantly decreased correlation during the running phase. Significance found in CN-VL mean correlations: *p(Eu) = 0.019, **p (TcMAC21) = 0.007 by paired t-test. **H** Discrimination Index (DI) for quantifying the changes in neural synchrony between stationary versus running phases. DI was defined as the difference in mean pairwise Pearson’s correlations of bulk activity between motor thalamus and cerebellar nuclei recordings during each locomotor epoch, divided by the sum of standard deviations (see METHOD). Data are presented as the mean ± SEM. ***p < 0.001 by Unpaired t-test.

Given our observations of disrupted cerebellar parasagittal organization and altered climbing fiber innervation patterns in TcMAC21 mice, we investigated whether these developmental alterations impair error signal processing through cerebello-thalamic pathways during motor behavior. Three cerebellar nuclei (fastigial, interposed, and lateral) conduct feedback and coordinate motor signals [84–87] through their projection to the ventral thalamus to modulate thalamo-cortical networks [88, 89]. Among these, we focused on the interposed nucleus, which we will refer to as cerebellar nuclei (CN) throughout this study. We reasoned that, by comparing the neural responses of CN and their downstream thalamic targets (ventrolateral nucleus of thalamus, VL) in TcMAC21 and euploid mice during voluntary locomotion, we can directly assess the impact of trisomy 21 on this critical motor feedback circuit. Identification of the glutamatergic CN neurons projecting to motor thalamus was achieved with retrograde Cre virus (rgAAV-Cre) injection in the VL to tag projection neurons with conditional GECI (FLEX.jRCaMP1b) expression in the CN (Fig. 5A; injections and optic fiber cannula implants validation in Supplementary Figure 5A, B). We implemented a free wheel-running task in head-fixed mice, allowing for precise tracking of movement states and velocity while simultaneously recording neural activity in the cerebellothalamic tract (CbT) from both CN and VL using multi-site fiber photometry [30, 90]. Due to the self-initiation nature of the task, we found variation between our mice in how motivated they were in engaging in locomotor activity; some engaged more in running bouts than others during a 25-min testing session. Using “percentage of time engaging locomotor activity” as the measure of motivation, we found no motivational difference between euploid and trisomy mice (Supplementary Figure 1D–F).

We observed bulk neural responses in both CN and VL that were tuned to the onset of wheel running and were generally disengaged during the stationary phases (velocity=0) (Fig. 5B, C; also see Supplementary Figure 5C–E). We quantified the area under the curve (AUC) of the ΔF/F trace and found no significant differences between euploid (n = 5 mice) and TcMAC21 (n = 6 mice) in the average activity of any of the brain regions (Fig. 5D; ANOVA with Sidak’s post-hoc test, cerebellar nuclei p = 0.2461, ventrolateral thalamus p = 0.4676). However, during the wheel-running, we found that euploid mice displayed significantly higher magnitude of ΔF/F (z-scored) in both VL and CN compared to stationary phase (Fig. 5E; Peak CN ΔF/F stationary vs. running, Eu: p = 0.0037, TcMAC21: p = 0.0592; Peak VL ΔF/F stationary vs. running, Eu: p = 0.0234, TcMAC21: p = 0.0109, Unpaired t-test with Welch’s correction); however, this locomotor state-dependent activity distinction was absent in TcMAC21 mice. We extracted dynamical parameters of CN and VL activities during initiation or disengagement from running, including: latency to peak mean ΔF/F, 10% rise/fall, and Pearson’s correlation of activity between simultaneously recorded neuronal traces, and analyzed these by principal component analysis (PCA) to reduce the dimensionality of the fiber photometry data. We found that the underlying neural dynamics captured by fiber photometry can segregate subjects based on genotype. The clusters of euploid and TcMAC21 mice had a centroid distance of 3.40 (Fig. 5F; PC1: 30.1% variance, PC2: 23.4% variance).

To determine whether locomotor state modulates the neural synchrony between cerebellar nuclei and motor thalamus, we calculated synchronicity during stationary and running phases. The increased CN-VL correlations (Pearson’s r during running epochs) were specific to euploid mice, while decreased CN-VL correlations were observed in TcMAC21 mice (Fig. 5G, euploid n = 5, TcMAC21 n = 6; Pearson’s correlation coefficients, Eu: p = 0.0197, TcMAC21: p = 0.0076, Paired t-test). To directly compare these between genotypes, we used a pattern discrimination index (DI), which measures how distinctly running and stationary states are reflected in inter-regional correlations (see METHOD). Euploid mice showed a DI of 0.3803, indicating robust state-dependent modulation of neural synchrony. In contrast, trisomy mice exhibited a significantly lower DI of -0.3797 (Fig. 5H, euploid n = 5, TcMAC21 n = 6; F_(5,4)_ = 1.804, p = 0.0007, Unpaired t-test), suggesting impaired locomotor-dependent coordination between these regions.

**Fig. 6:**
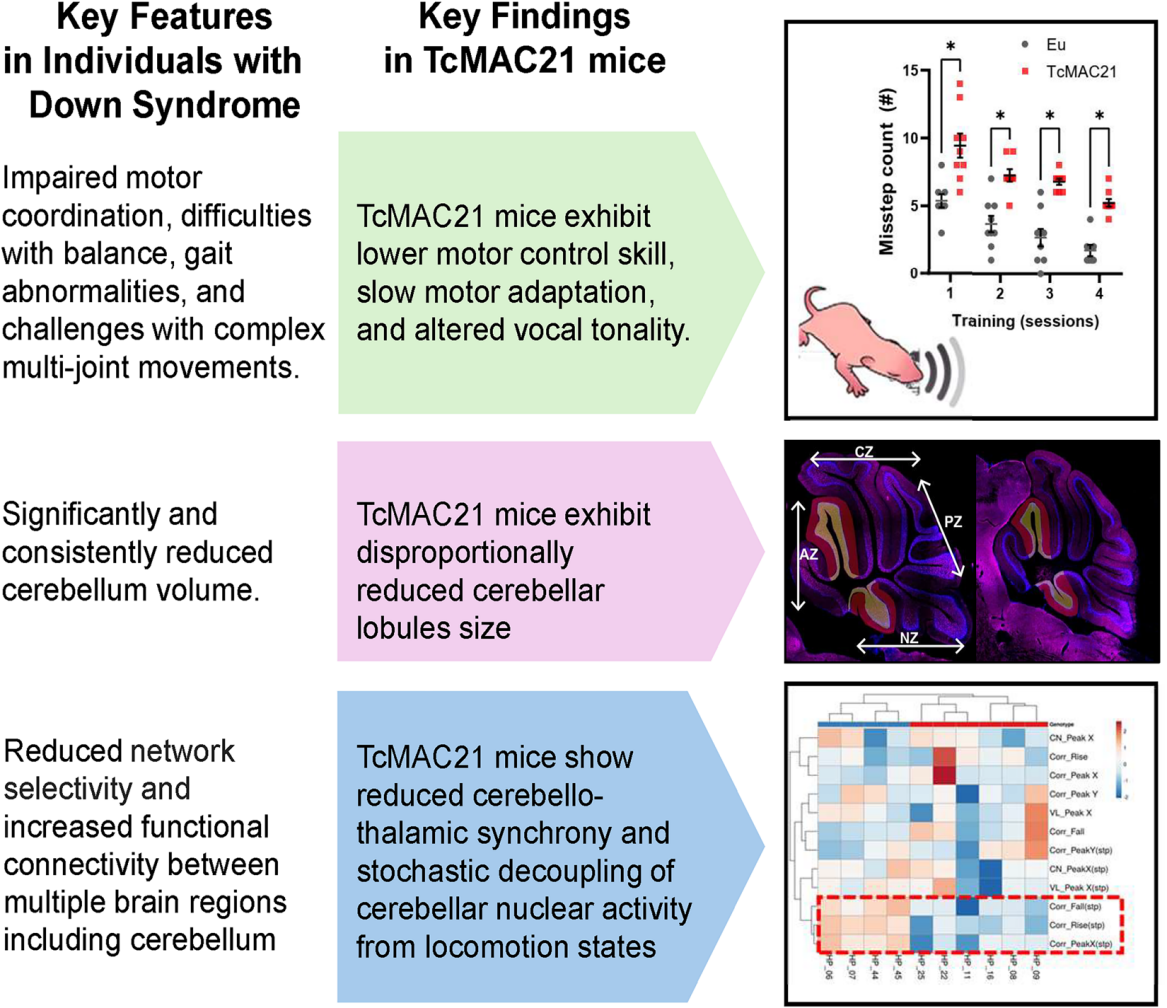
Comprehensive characterization establishes TcMAC21 as a model to study cerebellar circuit changes in Trisomy 21. Summary schematic integrating key findings: Partial list of key observations in individuals with DS (left) and our parallel findings in the TcMAC21 model (right), suggesting that the TcMAC21 model provides insights into the cerebellar basis for motor deficits, extending beyond the traditional focus on cortical dysfunction.

Collectively, our findings demonstrate that the TcMAC21 model recapitulates the core cerebellar pathophysiology observed in individuals with DS (Fig. 6). The model demonstrates strong validity across key areas: motor coordination deficits match clinical observations, cerebellar size reductions mirror human brain changes, and disrupted cerebello-thalamic circuits explain the underlying motor dysfunction. These results establish TcMAC21 as a robust platform for studying cerebellar dysfunction in DS.

## Discussion

We demonstrate that the TcMAC21 mouse model exhibits specific cerebellar-dependent motor and communication deficits that parallel human Down syndrome phenotypes. By employing the ErasmusLadder paradigm, which minimizes confounds from physical variables like hypotonia [91], we identified distinct impairments in inter-limb coordination and associative learning. These deficits mirror those seen in other cerebellar circuit mutations [28, 61] and *Nlgn3*^KO^ autism models [62], suggesting shared mechanisms of cerebellar dysfunction across neurodevelopmental disorders. Motor dysfunction and USV alterations are consistent features across multiple DS models [50, 92–94], highlighting the robustness of these phenotypes in modeling the human condition [22].

Our findings reveal a potential developmental mechanism whereby trisomy 21 disrupts climbing fiber-Purkinje neuron connectivity. The observed enlarged VGluT2 synapses and altered cerebellar nuclear output align with human imaging studies showing cerebellar afferent abnormalities in individuals with DS [95, 96]. These converging lines of evidence suggest that olivocerebellar circuit disruption is a conserved feature across species. The disrupted cerebello-thalamic connectivity we observe provides potential circuit-level mechanisms underlying the motor coordination and speech difficulties consistently observed in individuals with DS, offering new targets for therapeutic intervention during critical developmental windows. The vocal communication phenotype in TcMAC21 mice provides insight into the developmental origins of speech impairments in DS. The selective disruption of frequency modulation, rather than global vocalization deficits, suggests specific perturbations in circuits controlling vocal complexity [2, 97]. The bidirectional impairment in pup-dam communication further indicates that trisomy 21 affects both expressive and receptive aspects of social communication, consistent with clinical observations. Altogether, these findings suggest that early perturbations in cerebellar circuit organization may underlie both motor and communication deficits.

Our data revealed two key alterations in TcMAC21 mice compared to euploid controls: elevated VL thalamic responses and delayed ramping of thalamic activity during locomotor initiation. The significantly higher VL activity in TcMAC21 mice, quantified by normalized area under the curve during running epochs, suggests aberrant thalamic activation during locomotion. This hyperactivity may reflect either compensatory mechanisms or maladaptive responses due to impaired cerebellar nuclear modulation of VL neurons. Additionally, the increased latency from locomotor onset to peak VL activity in TcMAC21 mice indicates impaired temporal processing of motor-related signals in the thalamus. The altered temporal precision in cerebellar nuclear output during locomotor state transitions likely reflects improper integration of climbing fiber error signals, potentially arising from disrupted Purkinje neuron zonation patterns, altered climbing fiber synaptic properties, or both. Further circuit-specific manipulations will be required to dissect the relative contributions of these anatomical alterations to the observed physiological dysfunction.

These physiological recordings uncovered reduced cerebello-thalamic synchrony during motor behavior, providing a circuit-level mechanism for impaired motor learning. The ability to recapitulate both synaptic and behavioral phenotypes through developmental manipulation of Purkinje neuron calcium signaling establishes a causal link between early calcium dysregulation and persistent cerebellar dysfunction. Our findings suggest altered calcium homeostasis as a potential therapeutic target. Previous work by Schonewille et al. examining Purkinje neuron calcium signaling provides important mechanistic context for understanding these deficits [70]. The conditional knockout of calcium/calmodulin-activated protein phosphatase 2B in Purkinje neurons (L7-PP2B) demonstrates that disrupting calmodulin and calcineurin signaling profoundly impairs cerebellar learning. Remarkably, the L7-PP2B mice showed not only abolished parallel fiber LTP while preserving LTD, but also exhibited severe deficits in both gain increase and gain decrease adaptations of the vestibulo-ocular reflex, as well as impaired acquisition of conditioned eyeblink responses. These behavioral phenotypes exceeded those observed in kinase mutants with impaired LTD, suggesting that phosphatase-mediated potentiation may be even more critical for cerebellar motor learning than previously appreciated. The convergence of evidence from both gain-of-function in Down syndrome, where calcineurin is suppressed by HSA21 gene products, and loss-of-function in the L7-PP2B model underscores the fundamental importance of balanced calcium signaling for cerebellar circuit function. However, genetic manipulation of PP2B with assessment of adult parallel fiber plasticity and vestibulo-ocular reflex paradigms differs substantially from our postnatal chemogenetic approach during P9 to P21 and our focus on climbing fiber afferents and locomotion adaptation. We therefore consider these findings strongly supportive of the underlying mechanism while recognizing the developmental and circuit specificity of our phenotypes. Rescue experiments require careful consideration, as interventions that normalize cerebellar volume achieve only partial behavioral recovery, suggesting that the core pathology lies in altered synaptic integration rather than structural deficits alone [15]. While inhibitory DREADDs could potentially normalize climbing fiber morphology by reducing Purkinje neuron calcium influx, the complex interplay between development and calcium signaling suggests that combinatorial approaches targeting both pathways may be necessary for functional rescue. Future studies should rigorously test this therapeutic strategy.

We acknowledge several important limitations of the TcMAC21 model. As a transchromosomic rather than trisomic model, TcMAC21 expresses human genes (with potentially different regulatory elements and expression patterns) in a mouse cellular environment. While providing excellent genetic coverage of HSA21 (93% of protein-coding genes), this model is susceptible to potential instability through homologous recombination across generations, which may contribute to experimental variability. Another limitation is that we did not include females in the ErasmusLadder assay, as female TcMAC21 mice show size deficits that would confound the interpretation of step length and pattern at all stages of development in this assay. Given the primary goal of this study was to investigate genotype-dependence and the convergent sex-independent findings across structure, synapse, and motor learning behavior (i.e., rotarod test), and in the literature [98–100], restricting ErasmusLadder to males was a principled design choice that does not alter our mechanistic conclusions about cerebellar circuit dysfunction in DS. Despite these limitations, the TcMAC21 model offers valuable insights into cerebellar circuit dysfunction in DS by providing the most complete representation of HSA21 genes available, circumventing many limitations in genetic representation and phenotypic stability found in earlier models [26, 101].

The cerebellum is consistently and significantly reduced in individuals with DS [3, 4, 14, 102], aligning with their delayed motor development [103], poorer motor competence [39, 41], and impaired linguistic skills [104, 105]. Earlier DS genetic models showed variable results in motor assessments [23, 106], representing a significant challenge in the field for addressing underlying mechanisms of motor dysfunction. Here, we demonstrated that the TcMAC21 model offers opportunities to investigate how cerebellar circuit disruptions interact with other affected brain regions in DS. Future studies should examine the molecular pathways linking trisomy 21 to calcium dysregulation and explore therapeutic strategies targeting early cerebellar development.

## Supplementary information

SUPPLEMENTARY FIGURE TITLES AND LEGENDS

SI Figure 1

SI Figure 2

SI Figure 3

SI Figure 4

SI Figure 5

## Data Availability

All data supporting the findings of this study are available within the article and its supplementary information files. Additional data and code generated or analyzed are available from the corresponding author (KH) upon reasonable request.
