# Supplementary material for: Altered motor coordination, vocal communication, and cerebellar circuit connectivity in mice carrying a near-complete human chromosome 21
Source: Transl Psychiatry. 2025 Nov 22;16:14. doi: 10.1038/s41398-025-03744-2 (PMC12789671; doi:10.1038/s41398-025-03744-2)
Supplement: Supplementary file 1 — SUPPLEMENTARY FIGURE TITLES AND LEGENDS [file 41398_2025_3744_MOESM1_ESM.docx]

**Supplementary information (SI)**

Summary: The Supplementary Information (SI) figures in the manuscript illustrate control experiments and detailed methodological validations. **SI Figure 1** presents body measurements and behavioral controls for the ErasmusLadder task. **SI Figure 2** reports an additional motor learning paradigm and examines the effects of biological variables. **SI Figure 3** shows the protein levels of a subset of genes located on HSA21 in the TcMAC21 model. **SI Figure 4** provides a detailed analysis of climbing fiber patterns and validates the AAV delivery methods. **SI Figures 5 and 6** present supporting data for the multi-fiber photometry calcium recordings, including setup verification and representative example traces.

SUPPLEMENTARY FIGURE TITLES AND LEGENDS

**Supplementary Figure 1: Body Size Measurements and Behavioral Control Experiment on Trisomic and Euploid Mice for Erasmus Ladder** **Motor Performance Assay, Related to Figure 1**

(A and B) Femur and tibia bones of euploid and TcMAC21 male mice at 8-week-old. The schematic depiction of limb skeletal measurements in young male TcMAC21 and euploid littermates showing no significant differences in femur length (Eu: 13.87 ± 0.37 mm, TcMAC21: 13.57 ± 0.65 mm) or tibia length (Eu: 18.07 ± 0.42 mm, TcMAC21: 18.04 ± 0.69 mm). Differences were Not Significant by t-test: *p* = 0.7688.

(C) Body weight tracked over time shows reduced weight gain in TcMAC21 mice compared to euploid controls. Data are represented as means ± SEM. Weight differences were Not Significant by unpaired t-test: *p*>0.9999, *p*>0.9999, *p*>0.9999, *p*=0.1031, *p*=0.0643, *p*=0.1994, and *p*=0.0735 respectively for 3-week to 12-week-old. At 13-week and 14-week-old comparisons, weight differences were Significant: **p* = 0.0167 and **p* = 0.0446 respectively.

(D, E and F) Evaluation of open field behavior and locomotion in neurologically intact TcMAC21 and euploid littermates at 8-week-old. Mean total distance traveled in the open field revealed no significant differences between sexes or between genotypes, indicating that motor performance deficits in the ErasmusLadder task are not due to differences in baseline activity levels or motivation. Percentage of time spent in different zones of the open field arena shows comparable exploration patterns between genotypes, suggesting no anxiety-related behavioral differences that could confound motor learning assessments. Each data point represents one mouse, with solid symbols indicating males (upper panels) and open symbols indicating females (lower panels). Data are represented as means ± SEM. Euploid mice (male/female): n = 9 / 6 , TcMAC21 mice (male/female): n = 11 / 6; TcMAC21 male versus female mice: *p*(total distance traveled) = 0.3112, *p*(center time) = 0.2409, *p*(margin time) = 0.0846; Unpaired t-test.

(G and H) Schematic presentation of Pup retrieval test. Time spent retrieving Euploid vs. TcMAC21 pups into the nest was reported. All dams were euploid. The individual pups were graphed. The inset figure depicts the summarized trend, showing retrieval latency decreased in trisomy pups. *p*(1^st^)= 0.1299, **p*(2^nd^)= 0.0178, *p*(3^rd^)= 0.1938. Unpaired t-tests.

(I) Maternal behavior is impaired in TcMAC21 dams. The retrieval latencies of three pups on the of naive euploid and TcMAC21 dams are shown. There were significant differences between euploid and TcMAC21 dams in the retrieval latency of the third pup. Data are represented as means±SEM. *p*(1^st^)= 0.4284, *p*(2^nd^)= 0.0639, **p*(3^rd^)= 0.0049. Unpaired t-tests.
(J) Auditory cue was used to learn an active avoidance task. After weaning pups, the hearing of dams was evaluated using active avoidance paradigm. We found no difference in the percentage of trials where the mice correctly paired the cue-tone with shuttling between boxes to avoid the shock, comparing between female euploid and TcMAC21mice (F=1.326, *p*= 0.2800).

**Supplemental Figure 2. Sex-specific analysis of motor performance, cerebellar morphology, and synaptic characteristics in TcMAC21 mice, Related to Figure 1&2.**

(A) Left: Schematic of accelerating rotarod task. Middle: Rotarod performance showing latency to fall across training sessions for male (left) and female (right) mice, comparing euploid (Eu) and TcMAC21 groups. Right: Direct comparison of rotarod performance between male and female TcMAC21 mice across sessions. TcMAC21 mice of both sexes show significantly reduced latency to fall compared to euploid controls, with no significant difference between TcMAC21 males and females.

(B) Cerebellar morphological measurements and synaptic characteristics comparing euploid and TcMAC21 mice within each sex. Top row: Male comparisons showing molecular layer cross-sectional area, granule cell layer cross-sectional area, anterior zone measurements, and VGluT2 puncta size. Bottom row: Female comparisons showing the same parameters. Both sexes exhibit consistent reductions in cerebellar size and enlarged climbing fiber synapses in TcMAC21 mice.

(C) Sex comparison within TcMAC21 mice for cerebellar morphology (granule cell layer, molecular cell layer cross-sectional areas), synaptic measurements (VGluT2 puncta size, VGAT puncta size), and frequency distribution of VGluT2 immunoreactivity. No significant differences observed between males (M) and females (F) in TcMAC21 mice across all measures.

**Supplementary Figure 3: Western blots validate HSA21 gene–dosage amplification altering abundance calcium regulators in cerebellum at protein level.**

(A) Immunoblots of DYRK1A, regulator of calcineurin 1, phosphodiesterase 9A, and pericentrin protein (encoded by a subset of HSA21 genes and their mouse orthologs) in cerebellar lysates from adult (8 – 12 weeks old) euploid and TcMAC21 male (M) and female (F) mice. Proteins were separated on 10% SDS–PAGE and probed on nitrocellulose membranes. β-Actin (42 kDa) served as the loading control and showed no genotype- or sex-dependent change. TcMAC21 animals of both sexes exhibit a substantial increase in PDE9A, DYRK1A, and RCAN1 abundance. Additional blots for the high–molecular-weight centrosomal protein PCNT (resolved on 3–8% Tris–Acetate gels) show a moderate increase in TcMAC21 compared with Eu; vinculin (145 kDa) served as the high–molecular-weight loading control. (B) Representative cerebellar sagittal sections co-immunostained for PCP4 (cyan) and the Purkinje-neuron marker calbindin (magenta), showing stronger PCP4 signal in the Purkinje cell layer of TcMAC21 compared with euploid mice. Bottom: Quantification of PCP4 fluorescence within calbindin-positive Purkinje neurons (per-animal mean). Bars show mean ± SEM. with individual animals overlaid (n= 4 per genotype). Euploid: 295,920 ± 29,299 a.u.; TcMAC21: 648,938 ± 89,476 a.u.; ~2.2-fold increase in TcMAC21. Unpaired t-test: **p = 0.0095. Scale bar: 100 μm.

**Supplementary Figure 4: Analysis of Climbing Fiber Innervation Patterns, Related to Figure 2, and Validation of Systemic AAV Delivery for Purkinje neuron Targeting, Related to Figure 3.**

(A) Left: Representative sagittal cerebellar sections showing VGluT2-positive climbing fiber terminals (magenta) in the molecular layer of euploid and TcMAC21 cerebella. Right: High-res histology stack of presynaptic marker and 3D rendering of pre-synapse areas (Blue, excluded puncta; Yellow, assigned puncta). Scale bar: 50 μm and 5 μm, respectively.

(B and C) Quantification of VGluT2-positive climbing fiber extension in the molecular layer was reduced in TcMAC21 mice, measured as the molecular layer height reached by VGluT2-positive terminals. (C) Analysis of VGluT2 puncta along the dendritic arbors of Purkinje neurons. Analysis showed Significant decreased in both height and width. Data are represented as means ± SEM. ***p = 0.0009, **p = 0.5532, respectively; n = 9 for euploid cerebella, n = 11 for TcMAC21 cerebella, t-test.

(D) Schematics of the surgical approaches for Intra-Cisterna Magna (ICM) injection cre- dependent DREADD AAV virus for restricted Purkinje neurons expression during postnatal period.

(E) Representative images showing widespread viral transduction following systematic delivery of AAV.CAG.FLEX-hM3D-CTS (1x10^10 VG per animal, labelled by co-expression of RFP in Red) via ICM delivery at P2. Scale bar: 500 μm.

(F) Higher magnification (left) and whole brain (right) images showing specific expression in Purkinje neurons with complete cell morphology labeling including dendrites and axons, without expression outside the cerebellum. Quantification of viral targeting specificity showing 66.87 ± 3.36 % of Calbindin-positive Purkinje neurons co-expressed RFP in lobule X. Scale bar: 100 μm (left), 1 mm (right).

(G) Topology of tyrosine hydroxylase expression between euploid and TcMAC21 mice in lobule IX. Coronal sections immunolabeled for tyrosine hydroxylase (green) demonstrate that tyrosine hydroxylase-positive Purkinje neurons are topographically distributed in parasagittal stripes (arrowheads), following a pattern similar to that of HSP25-positive Purkinje neurons previously reported in the literature ^81,82^. In contrast, TcMAC21 mice exhibit disrupted parasagittal patterning of tyrosine hydroxylase-positive neurons, indicating altered cerebellar zonation. Scale bar = 500 μm.

**Supplementary Figure 5: Supporting Data for Multi-Fiber Photometry Calcium Recordings, Related to Figure 5**

(A) Schematic of a mouse brain coronal section showing jRCaMP1a reporter expression from cerebellar nuclei (CN) and ventrolateral thalamus (VL). Asterisk designates sites of viral injection (CN: AP -6.13 mm, ML ±1.60 mm, DV -3.60 mm; VL: AP -0.9 mm, ML ±1.00 mm, DV -3.75 mm relative to bregma). Restricted reporter expression spread was shown as overlapping shaded area, n = 11 animals.

(B) Representative histological verification of fiber optic cannula placements. VL (left panel) and CN (right panel) targeting showing fiber tract above confined jRCaMP1a expression (red) area. DAPI counterstain in blue. Brain region abbreviations -M1: Primary Motor Cortex, M2: Secondary Motor Cortex, LV: Lateral Ventricle, HP: Hippocampus, 3V: Third Ventricle, Str: Striatum, RT: Reticular Thalamic Nucleus, VL: Ventrolateral Thalamic Nucleus, VA: Ventral Anterior Thalamic Nucleus, Re: Reuniens Thalamic Nucleus, HY: Hypothalamus, FN: Fastigial Nucleus (of the cerebellum), IPN: Interposed Nucleus (of the cerebellum), V Cb: Lobule V of the Cerebellar Vermis. Scale bars: 1000 μm.

(C) Example traces showing simultaneous calcium signals (mean ΔF/F) recorded from an euploid mouse in CN (red) and VL (green) during the transition (t=0) from stationary to running periods.

(D) Example traces from a euploid mouse showing increased synchronicity between CN and VL calcium signals during locomotor phase compared to stationary phase.

(E) Example traces from a TcMAC21 mouse showing decreased temporal synchronicity during running phase compared to euploid.
