# Supplementary figures and images for: Altered motor coordination, vocal communication, and cerebellar circuit connectivity in mice carrying a near-complete human chromosome 21
Source: Transl Psychiatry. 2025 Nov 22;16:14. doi: 10.1038/s41398-025-03744-2 (PMC12789671; doi:10.1038/s41398-025-03744-2)

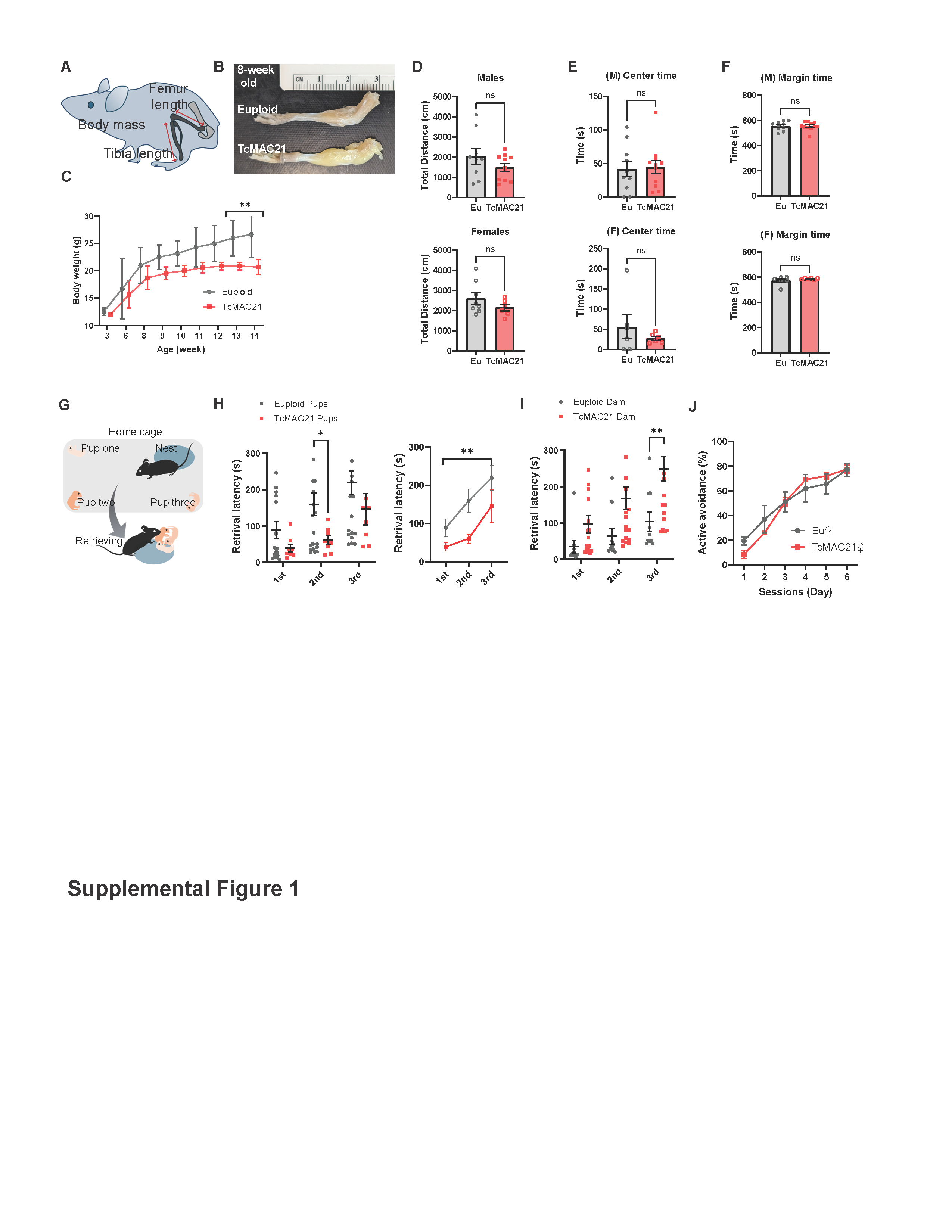

Supplement: Supplementary file 2 — SI Figure 1 [file 41398_2025_3744_MOESM2_ESM.tif]

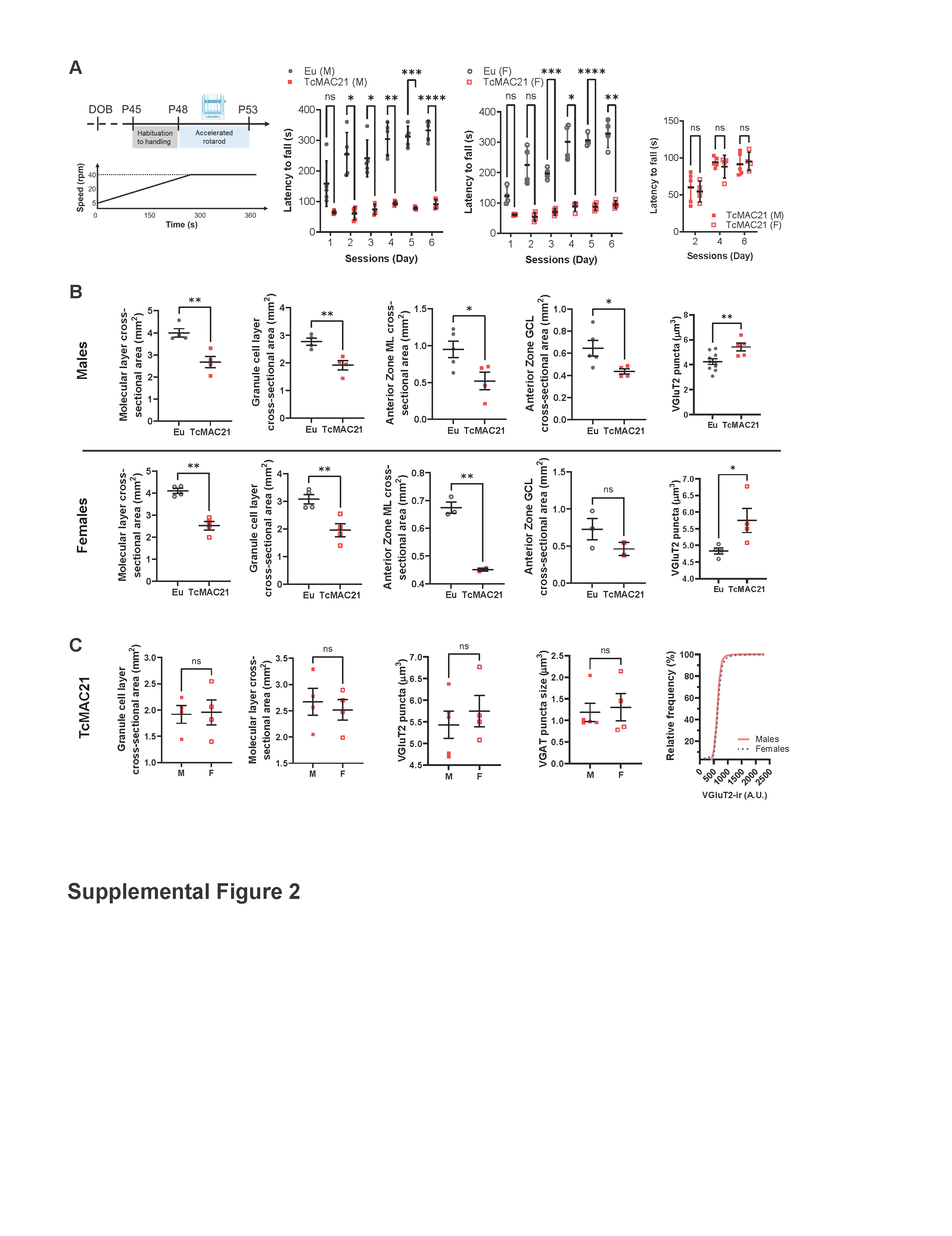

Supplement: Supplementary file 3 — SI Figure 2 [file 41398_2025_3744_MOESM3_ESM.tif]

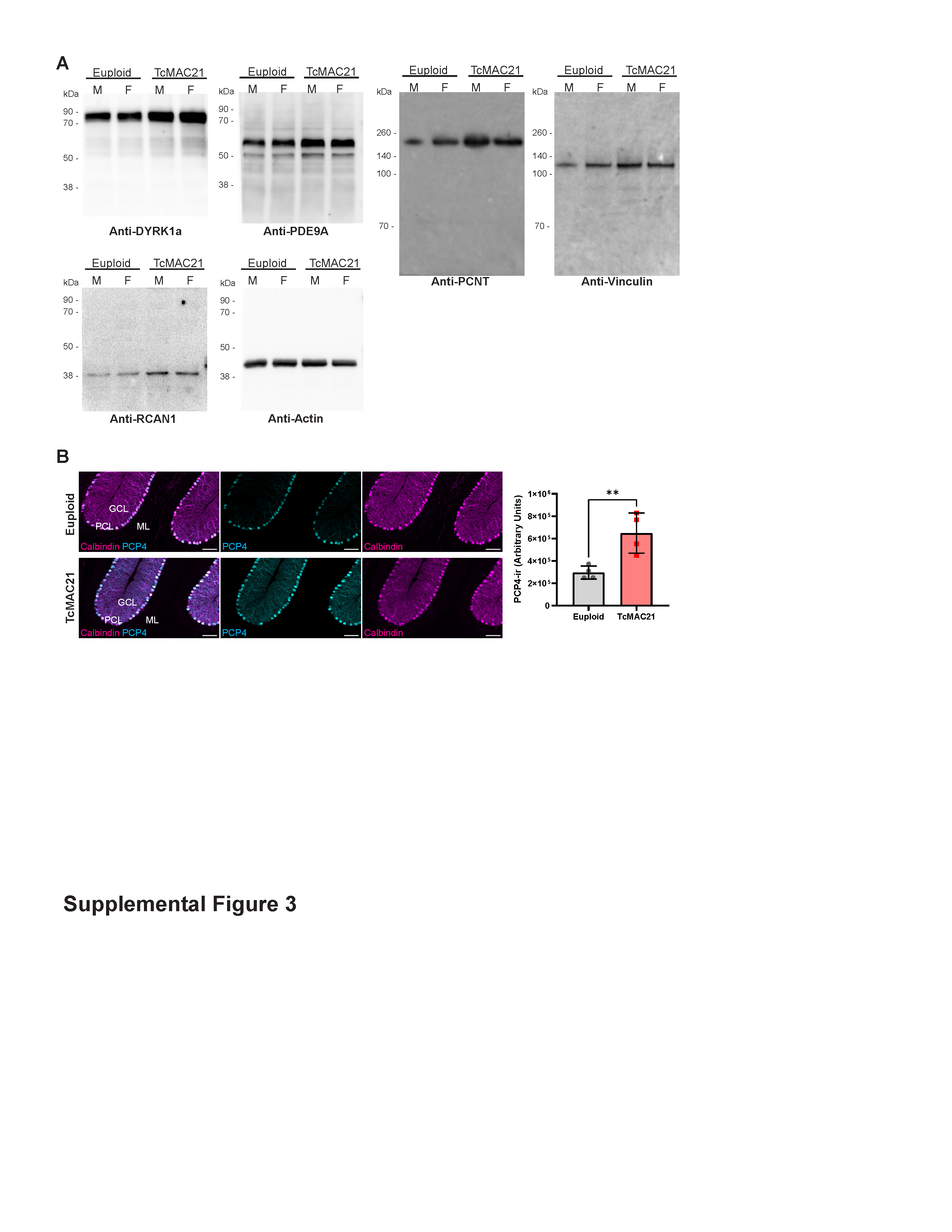

Supplement: Supplementary file 4 — SI Figure 3 [file 41398_2025_3744_MOESM4_ESM.tif]

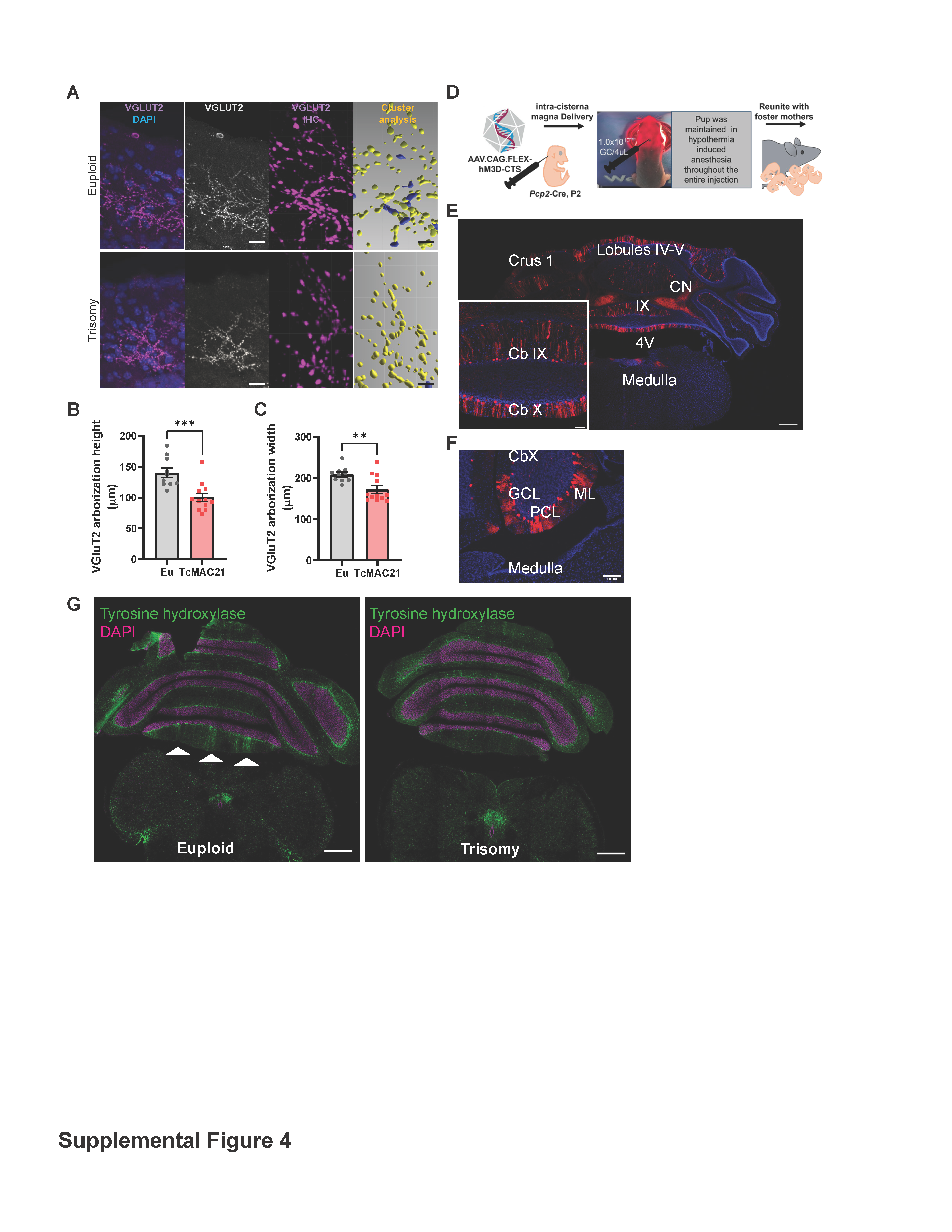

Supplement: Supplementary file 5 — SI Figure 4 [file 41398_2025_3744_MOESM5_ESM.tif]

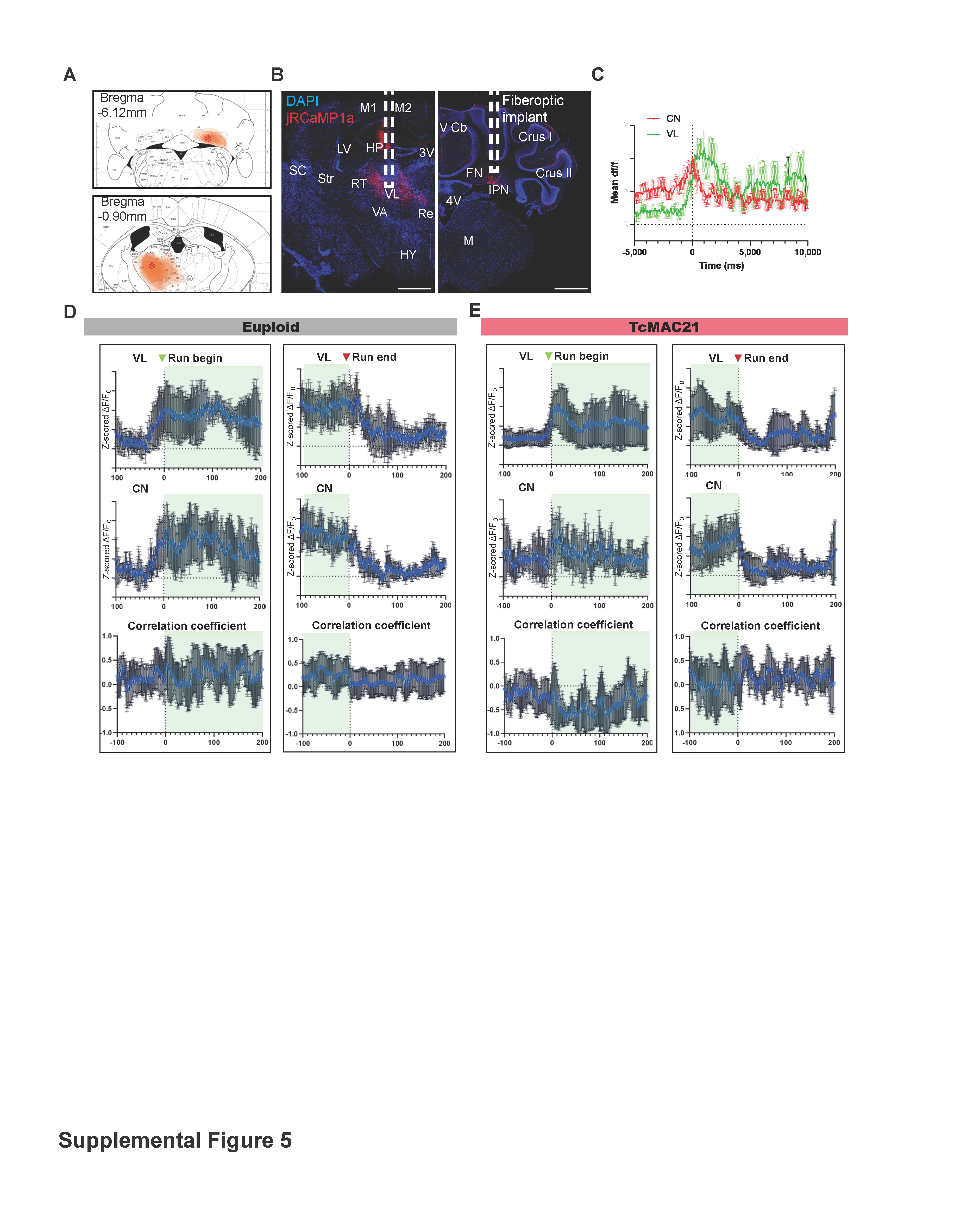

Supplement: Supplementary file 6 — SI Figure 5 [file 41398_2025_3744_MOESM6_ESM.tif]
